# BCL6B expression in hepatocellular carcinoma and its efficacy in the inhibition of liver damage and fibrogenesis

**DOI:** 10.18632/oncotarget.3857

**Published:** 2015-04-27

**Authors:** Weilin Wang, Pengfei Huang, Panyisha Wu, Rong Kong, Jiang Xu, Lufei Zhang, Qifan Yang, Qingsong Xie, Linshi Zhang, Xiaohu Zhou, Linghui Chen, Haiyang Xie, Lin Zhou, Shusen Zheng

**Affiliations:** ^1^ Key Lab of Combined Multi-Organ Transplantation, Ministry of Public Health, Hangzhou 310003, China; ^2^ Division of Hepatobiliary and Pancreatic Surgery, Department of Surgery, First Affiliated Hospital, Zhejiang University School of Medicine, Hangzhou 310003, China; ^3^ Collaborative Innovation Center for Diagnosis and Treatment of Infectious Diseases, Hangzhou 310003, China

**Keywords:** BCL6B, hepatocellular carcinoma, liver fibrosis, prognostic biomarker

## Abstract

B cell CLL/lymphoma 6 member B (BCL6B) is expressed in many normal tissues but expressed at very low levels in cancer tissues. It was reported that BCL6B inhibits hepatocellular carcinoma (HCC) metastases, but the exact role of BCL6B in HCC remains to be investigated. BCL6B expression was significantly decreased in HCC tissues compared with paired non-cancer tissues. Low BCL6B expression in tumors was correlated with shorter overall survival in patients, and multivariate Cox regression analysis revealed that BCL6B expression was an independent prognostic factor for human HCC patients. Moreover, a positive correlation between BCL6B expression and hepatic cirrhosis was found in an analysis of HCC clinicopathological characteristics. BCL6B expression was increased in rat fibrotic liver samples in response to liver injury. BCL6B transgenic rats were less susceptible to hepatocellular damage, inflammation and fibrosis. *In vitro* studies demonstrated that BCL6B inhibited the activation of hepatic stellate cells though upregulation of hepatocyte growth factor. In addition, transcriptomic microarray analysis was performed to explore the mechanisms in which BCL6B confers protection from tumorigenesis. In conclusion, BCL6B plays a pivotal role as a prognostic biomarker for HCC, and the restoration of BCL6B may be a novel strategy as an anti-fibrogenic therapy for human HCC.

## INTRODUCTION

Hepatocellular carcinoma (HCC) is the sixth most common malignancy worldwide and exhibits the third highest mortality rate. More than 90% of HCCs worldwide develop from a cirrhotic background as a result of chronic hepatitis B or C infection, alcohol abuse, or metabolic syndrome. Most of these cases will die from this complication, since the 5-year survival rate remains a dismal 12% with the currently available therapies [1, 2]. Therefore, novel treatments for liver cancer are urgently needed [3, 4]. For example, immunotherapy and potential novel gene therapy show good prospects for the treatment of HCC [5, 6].

Liver fibrosis is a common pathological process resulting from various chronic hepatic injuries, characterized by remodeling of the extracellular matrix (ECM) and excessive deposition of collagen. The activation of quiescent hepatic stellate cells (HSCs) into a myofibroblast-like phenotype is considered the pivotal event in the pathogenesis of liver fibrosis. ECM synthesis and deposition are regulated by many factors [7, 8]. The most well-known profibrogenic mediators expressed by activated HSCs include transforming growth factor beta (TGF-β) and platelet-derived growth factor, whereas hepatocyte growth factor (HGF) functions as an anti-fibrogenic mediator [9, 10] Although the effects of HGF in reducing the accumulation of ECM and the development of hepatic fibrosis have been investigated extensively, the exact mechanism regulating HGF expression in HSCs in response to liver injury and inflammation remains unknown.

BCL6B, also known as BAZF, plays a role in the nucleus as a sequence-specific transcriptional repressor [11]. Human BCL6B mRNA is expressed ubiquitously in human tissues, with abundant expression in the heart and placenta [12] BCL6B is required for the secondary responses of memory CD8^+^ T cells and mediates VEGFR and Notch cross-signaling in angiogenesis via the cullin3-based E3 ligase complex [13, 14]. BCL6B plays a pivotal role as a potential tumor suppressor in gastric cancer, and promoter hypermethylation, as a plasma DNA biomarker, is associated with poor survival of gastric cancer patients [15–17]. It was reported that ectopic expression of BCL6B resulted in significant suppression of HCC cell proliferation, invasion and metastasis *in vitro* and in mice, but the exact functional role of BCL6B in HCC remains to be investigated [18].

In the present study, we observed that BCL6B expression was significantly decreased in HCC tissues compared with paired non-cancer tissues. Moreover, a positive correlation between BCL6B expression and hepatic cirrhosis was found in an analysis of HCC clinicopathological characteristics. The causal relationship and physiological role of BCL6B in the development of liver fibrosis must be elucidated to demonstrate the role played by BCL6B in HCC.

## RESULTS

### Reduced BCL6B expression in HCC versus non-cancer tissues

**Figure 1:**
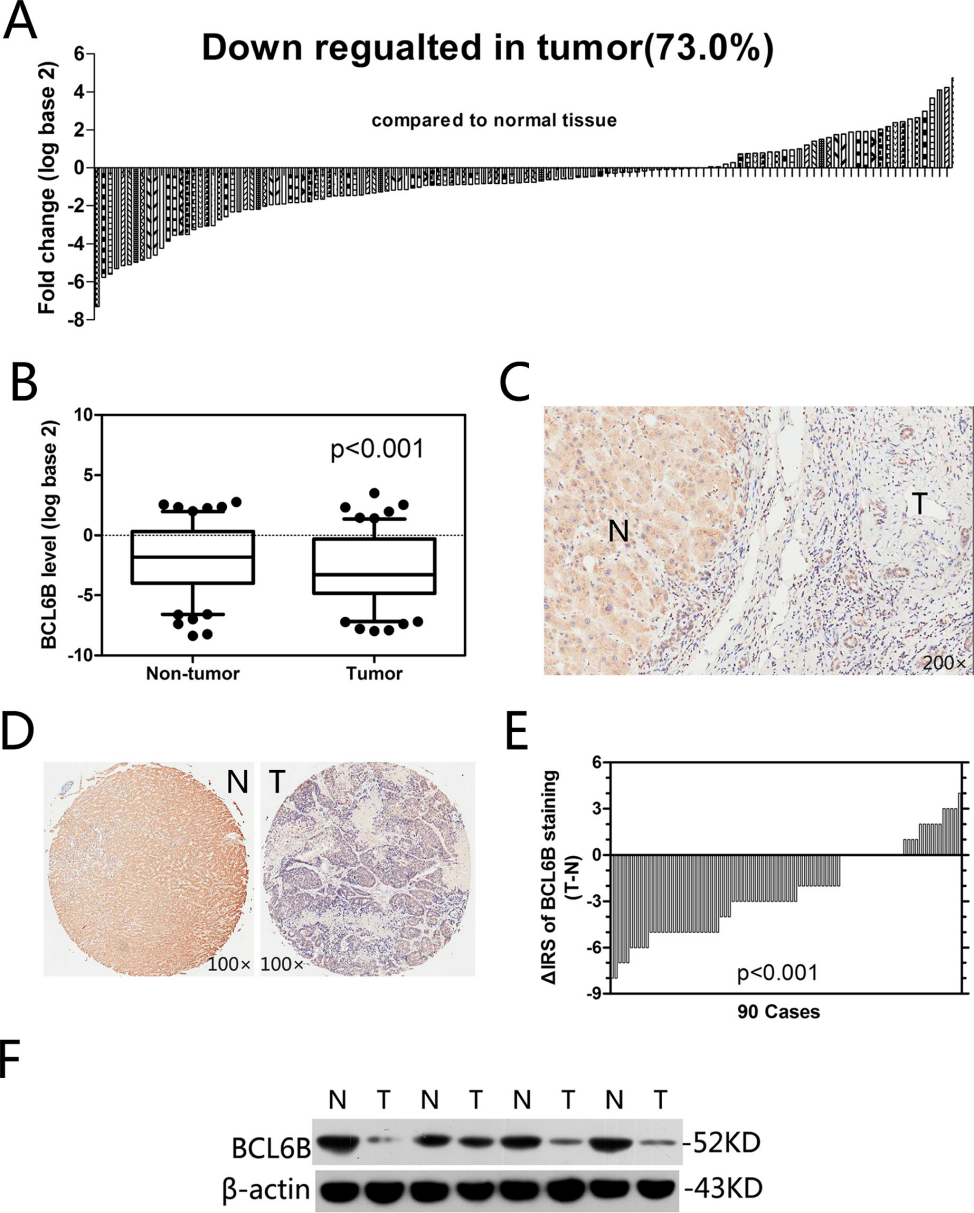
Relative BCL6B expression levels in HCC tissues **A.** In the vast majority of cancer tissues (73.0%), the BCL6B mRNA level was decreased. **B.** In all 126 tissue pairs, the downregulation of BCL6B expression in tumors was significant compared with adjacent non-cancer tissues. **C.** Representative images of immunohistochemical tissue staining using a BCL6B antibody. N, adjacent normal tissues; T, hepatic tumor tissues. Original magnification, × 200. **D.** Immunohistochemical tissue microarray staining using a BCL6B antibody; original magnification, × 100. N, adjacent normal tissues; T, hepatic tumor tissues. **E.** The distribution of the difference in BCL6B staining (ΔIRS= IRS_T_ – IRS_N_). The immunoreactivity score (IRS) for BCL6B staining was available for 90 tissue pairs; *p* values were calculated using the Wilcoxon test. **F.** BCL6B protein downregulated was confirmed by Western blot analysis. N, adjacent normal tissues; T, hepatic tumor tissues.

First, we analyzed the expression of BCL6B by quantitative PCR, Western blot and immunohistochemistry. In paired primary cancer and adjacent non-cancer tissues from 126 HCC patients (quantitative PCR cohort), 92 cases (73.0%) exhibited significant downregulation of BCL6B in cancer tissues (Figure 1A). In all 126 tissue pairs, BCL6B expression was significantly lower in tumors compared with adjacent non-cancer tissues (*p* < 0.001; Figure 1B). Immunohistochemical staining of the HCC tissues showed predominant localization of the BCL6B protein in the cytoplasm in the adjacent non-tumor tissues but significant downregulation in HCC tissues (Figure 1C). These results were confirmed in TMAs from 90 HCC patients, which showed a significant decrease in BCL6B expression in 69 of 90 HCC tissue samples (76.7%) compared with matched normal liver tissues (*p* < 0.001; Figure 1D, 1E). The analysis of BCL6B protein levels by Western blot confirmed the immunohistochemical results (Figure 1F).

### BCL6B expression is correlated with the clinicopathological characteristics and shorter survival of HCC patients

As shown in Table 1, BCL6B protein expression in cancer tissues from the quantitative PCR cohort was significantly correlated with clinicopathological features such as hepatitis B infection, liver cirrhosis and portal vein tumor thrombus (PVTT) (*p* < 0.05 for all). Moreover, the correlation between BCL6B expression and liver cirrhosis and PVTT was confirmed in the HCC TMA cohort.

**Table 1:** Relationship between expression levels of BCL6B and demographic and clinicopathological features of the individuals in two cohorts of HCC

Variables	Quantitative PCR cohort (126 cases)			TMAs cohort (90 cases)		
	Low (%)	High (%)	*p* Value	Low (%)	High (%)	*p* Value
All patients	92 (73.0)	34 (27.0)		58 (64.4)	32 (35.6)	
Age (years)						
< 50	25 (27.2)	7 (20.6)	0.499	14 (24.1)	7 (21.9)	1.000
≥ 50	67 (72.8)	27 (79.4)		44 (75.9)	25 (78.1)	
Gender						
Female	16 (17.4)	4 (11.8)	0.586	8 (13.8)	5 (15.6)	1.000
Male	76 (82.6)	30 (88.2)		50 (86.2)	27 (84.4)	
Hepatitis B						
Absent	28 (30.4)	4 (11.8)	0.038*	17 (29.3)	5 (15.6)	0.202
Present	64 (69.6)	30 (88.2)		41 (70.7)	27 (84.4)	
Hepatitis C						
Absent	91 (98.9)	33 (97.1)	1.000	57 (98.3)	32 (100)	1.000
Present	1 (1.1)	1 (2.9)		1 (1.7)	0 (0)	
Liver cirrhosis						
Absent	43 (45.7)	5 (14.7)	0.001*	29 (50.0)	7 (21.9)	0.013*
Present	49 (54.3)	29 (85.3)		29 (50.0)	25 (78.1)	
Preoperative AFP level (ng/mL)						
< 400	57 (62.0)	23 (67.6)	0.678	31 (53.4)	21 (65.6)	0.373
≥ 400	35 (38.0)	11 (32.4)		27 (46.6)	11 (34.4)	
Histopathologic grading						
Well + moderately	38 (41.3)	19 (55.9)	0.162	23 (39.7)	20 (62.5)	0.048*
Poorly	54 (58.7)	15 (44.1)		35 (60.3)	12 (37.5)	
Tumor size (cm)						
< 5	33 (35.9)	13 (38.2)	0.837	22 (37.9)	15 (46.9)	0.503
≥ 5	59 (64.1)	21 (61.8)		36 (62.1)	17 (53.1)	
Tumor number						
Single	69 (75.0)	25 (73.5)	1.000	41 (70.7)	21 (65.6)	0.641
Multiple	23 (25.0)	9 (26.5)		17 (29.3)	11 (34.4)	
PVTT						
Absent	65 (69.1)	31 (91.2)	0.018*	46 (79.3)	31 (96.9)	0.028*
Present	27 (30.9)	3 (8.8)		12 (20.7)	1 (3.1)	

* *p* < 0.05. The significance of the difference between groups in the table was assessed by Chi-squared tests (two sided Fisher's exact test). TMAs, tissues microarrays; AFP, alpha-fetoprotein; PVTT, portal vein tumor thrombus. HCC, hepatocellular carcinoma.

**Figure 2:**
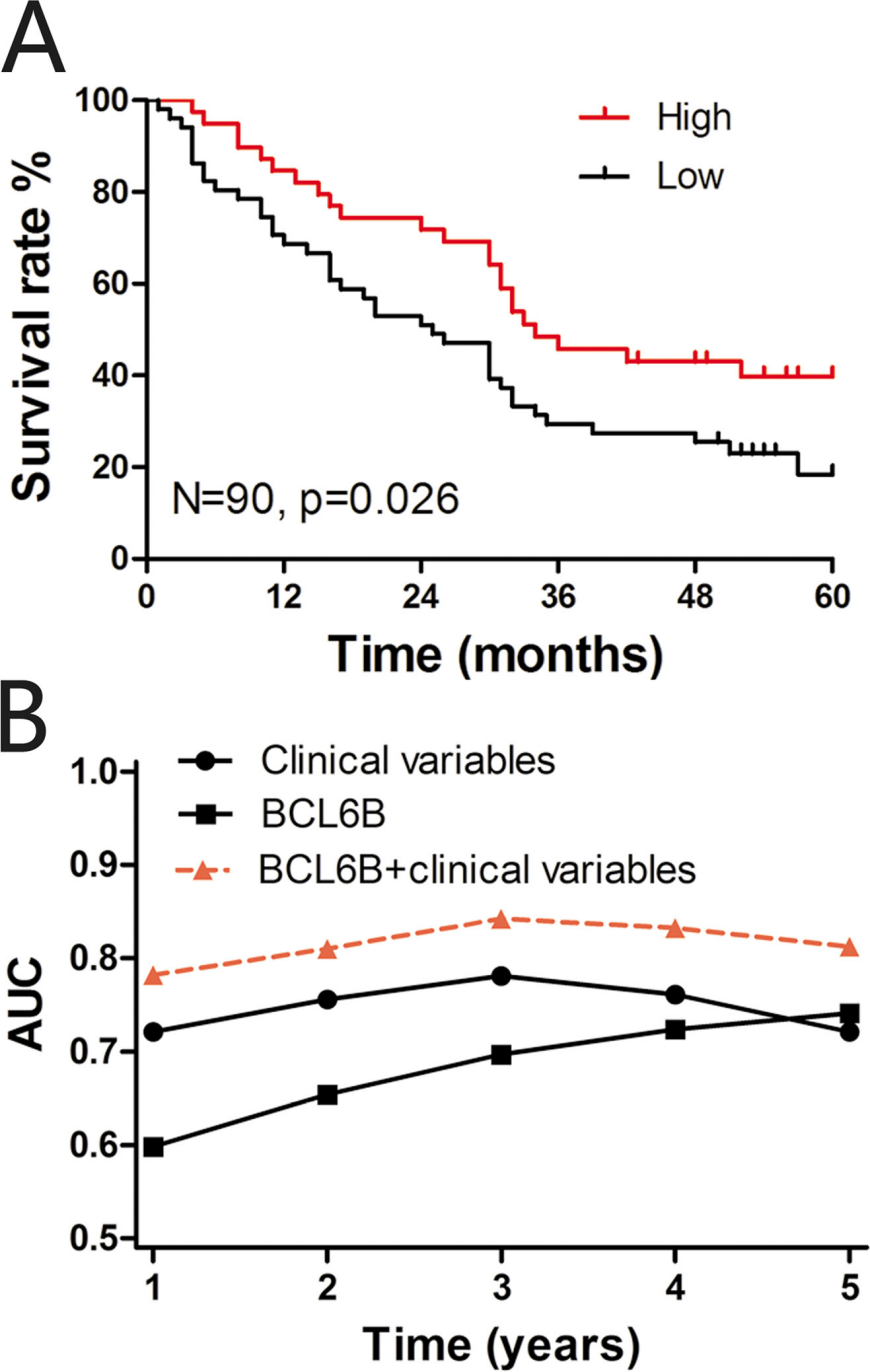
A. Kaplan-Meier curves depicting overall survival according to the BCL6B expression pattern in the tissue microarray (TMA) cohort (*n* = 90) The *p* values were calculated using the log-rank test. **B.** Time-dependent receiver operating curve (ROC) analyses. Figures show the time-dependent ROC analysis for the clinical risk score (TNM stage, histopathologic grading and preoperative AFP level), BCL6B risk score, and the combined BCL6B and clinical risk scores in the TMA cohort. AUC, area under the curve.

In the TMA cohort, Kaplan-Meier analysis revealed a significant correlation between low BCL6B staining and a poor overall 5-year survival in all HCC patients (*p* = 0.026, log rank test; Figure 2A). The median survival time of those patients with a high BCL6B expression score of their primary HCC was 34 months, whereas low BCL6B expression correlated with a shortened median survival time of 25 months. Next, multivariate Cox regression analysis indicated that BCL6B expression was an independent positive prognostic factor (RR = 0.459, 95% CI 0.252–0.834; *p* = 0.011; Table 2). As expected, the tumor stage and histopathological grading were also significant prognostic factors (*p* < 0.001 and *p* = 0.009, respectively).

**Table 2:** Multivariate Cox regression analysis of BCL6B and clinical variables predicting survival in HCC

Variables*	TMAs cohort (90 cases)	*p* value
	RR (95% CI)	
Age	1.002 (0.972–1.033)	0.883
Gender	0.848 (0.397–1.814)	0.671
TNM stage	2.772 (1.867 to 4.117)	< 0.001
Preoperative AFP level	1.516 (0.894–2.572)	0.123
Histopathologic grading	2.526 (1.261–5.060)	0.009
BCL6B expression	0.459 (0.252–0.834)	0.011

* Coding of variables: gender was coded as 0, male and 1, female; TNM stage was coded as 1, stage I/II and 2, stage III/IV; preoperative AFP level was coded as 0, < 400 ng/mL and 1, ≥ 400 ng/mL; histopathologic grading was coded as 0, well + moderately and 1, poorly; BCL6B expression was coded as 0, low and 1, high.

BCL6B, B cell CLL/lymphoma 6 member B; AFP, alpha-fetoprotein; TMAs, tissue microarrays; HCC, hepatocellular carcinoma.

To further evaluate the predictive ability of BCL6B expression, we conducted time-dependent ROC analysis, which indicated that in both cohorts the combination of the clinical risk score (Tumor Node Metastasis (TMN) stage, histopathologic grading and preoperative alpha fetoprotein (AFP) level) and BCL6B risk score had a markedly greater contribution than did either score alone. In the TMA cohort, the AUC at year 5 was 0.721 (95% CI 0.532 to 0.804) for the clinical risk score, and this was significantly increased to 0.812 (95% CI 0.705 to 0.878) when the clinical and BCL6B risk scores were combined (Figure 2B).

### BCL6B ameliorates hepatocellular damage and inflammation in response to liver injury

**Figure 3:**
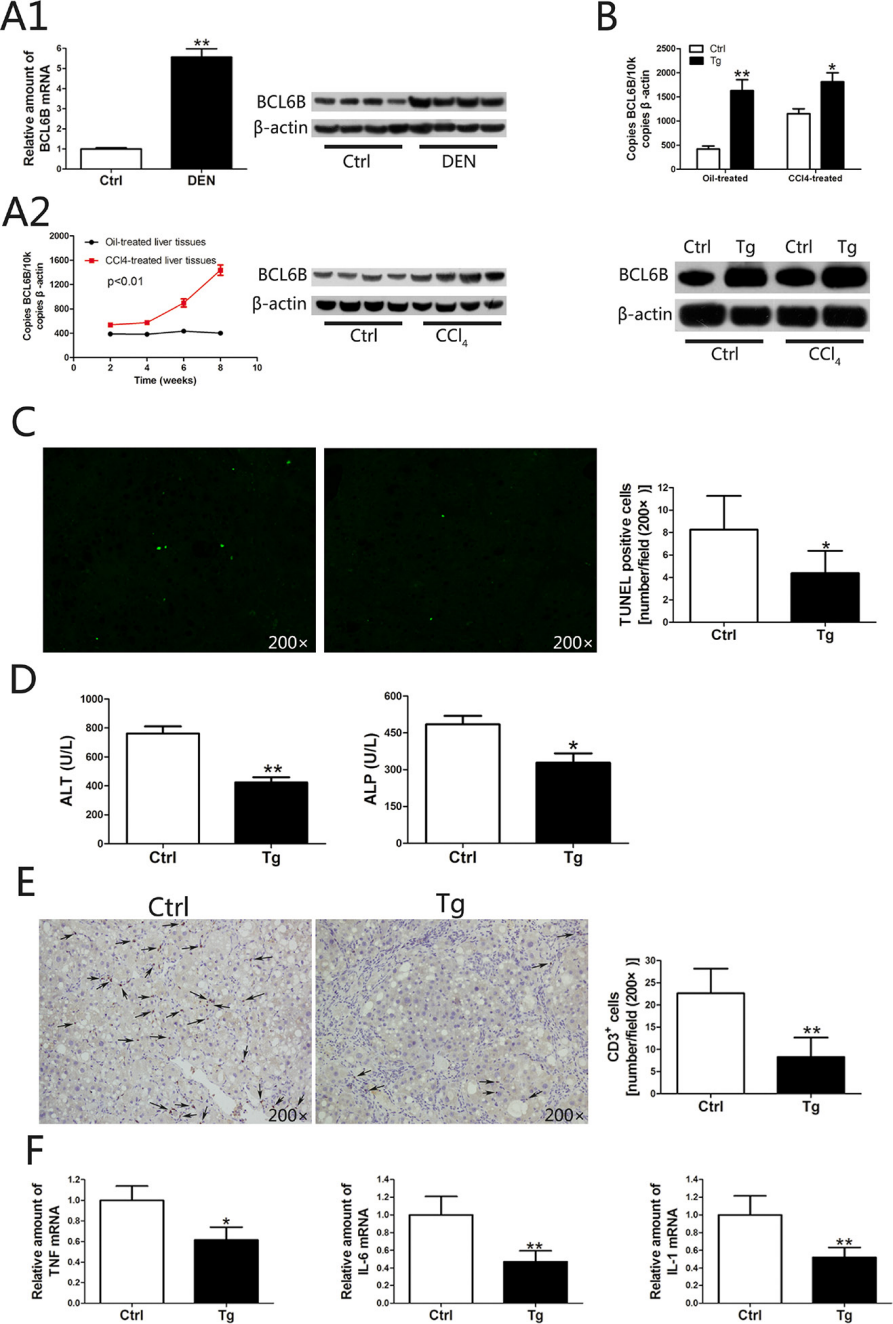
The function of BCL6B in inhibiting hepatocellular damage and inflammation **A.** Analysis of hepatic BCL6B mRNA and protein expression levels in rats in response to A1. acute liver injury after diethylnitrosamine (DEN) treatment for 48 h and A2. chronic liver injury after CCl_4_ treatment for 8 weeks (*n* = 8/group), compared with control rats. **B.** Effective overexpression of BCL6B in rat liver tissues. Absolute quantification of rat BCL6B mRNA expression by quantitative PCR of 2 μg cDNA from the livers of BCL6B transgenic (Tg) or control rats treated with corn oil or CCl_4_ 8 weeks after lentiviral injection (*n* = 6/group). **C.** Evaluation of apoptotic cells in liver tissues using the terminal deoxynucleotidyl transferase dUTP nick end labeling (TUNEL) assay in BCL6B Tg (*n* = 4) and control (*n* = 4) rats after CCl_4_ treatment for 8 weeks. **D.** Quantification of alanine aminotransferase (ALT) and alkaline phosphatase (ALP) serum levels in CCl_4_-treated BCL6B Tg and control rats. **E.** The number of CD3-positive inflammatory cells in the livers of control and BCL6B Tg rats after CCl_4_ treatment for 8 weeks. **F.** Hepatic mRNA expression of TNF, IL-6, and IL-1 in control and BCL6B Tg rats after CCl_4_ treatment for 8 weeks. *p < 0.05, **p < 0.01.

In humans, 70–90% of HCC cases are associated with advanced liver fibrosis or cirrhosis [19]. HCC arises most often in the presence of chronic liver inflammation and fibrosis/cirrhosis that potentially results from disturbances in metabolism, toxic insults or viral infection [20]. To investigate the positive correlation between BCL6B expression and hepatic cirrhosis in two cohorts of HCC patients (*p* = 0.001, *p* = 0.013; Table 1), we established rat models of liver injury under pathophysiological conditions. In the ALI model, the toxicity of DEN in the liver caused a marked upregulation of hepatic BCL6B expression in wild-type (WT) rats (Figure 3A1). In the CLI model, a gradual elevation of BCL6B mRNA was observed during the 8-week CCl_4_ treatment (Figure 3A2). Because lentiviruses infect most cell types and have been widely used for gene delivery in the liver, we evaluated the effect of BCL6B on ameliorating hepatocellular damage and inflammation using liver-specific BCL6B transgenic (Tg) rats, which express rat BCL6B mRNA in the liver at levels nearly fourfold higher than those in WT rats (Figure 3B). Although similarities in liver morphology were seen (Supplementary Figure 1), BCL6B Tg rats displayed a significantly lower number of apoptotic cells (Figure 3C), lower levels of serum transaminases and alkaline phosphatase (ALP; Figure 3D), and a lower number of recruited inflammatory cells (Figure 3E), compared with control rats, in response to hepatic injury via CCl_4_. These results were consistent with the reduced levels of proinflammatory genes (Figure 3F and Supplementary Figure 2) and fewer areas of necrosis (Supplementary Figure 3) in the livers of CCl_4_-treated BCL6B Tg rats. In contrast, BCL6B Tg and control rats showed no significant difference in the infiltration of inflammatory or apoptotic cells under the no-treatment conditions. In addition, the levels of serum transaminases, ALP and hepatic proinflammatory gene expression were similar between BCL6B Tg and control rats (Supplementary Figure 4).

### BCL6B attenuates liver fibrosis

**Figure 4:**
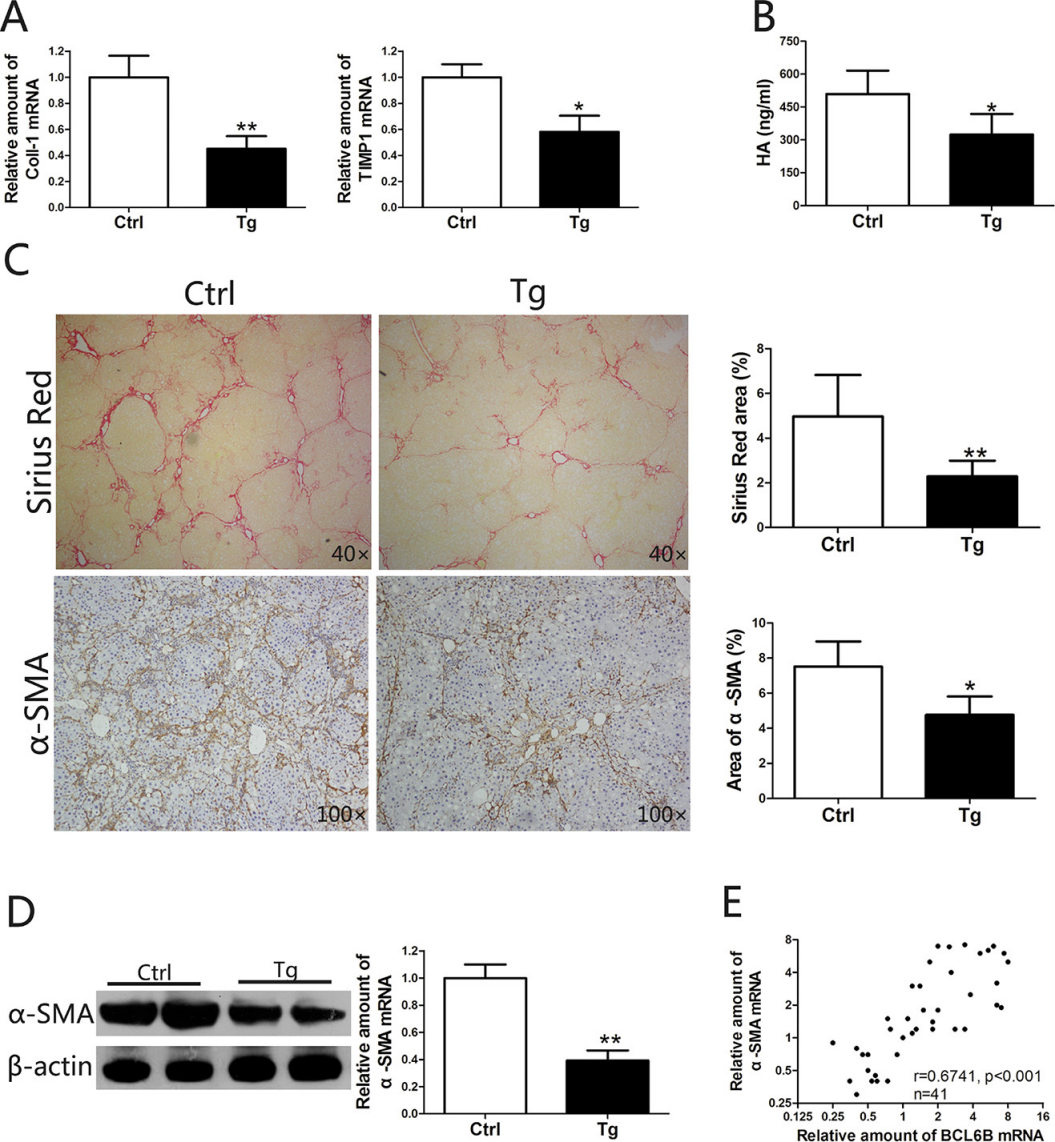
BCL6B attenuates liver fibrosis Sprague–Dawley rats (control) and BCL6B Tg rats (*n* = 8) were treated with CCl_4_ for 8 weeks. **A.** Expression of Coll-1 and TIMP1 mRNA in liver tissues of CCl_4_-treated control and BCL6B Tg rats. **B.** The hyaluronic acid (HA) levels in CCl_4_-treated control and BCL6B Tg rats are shown. **C.** Histological characterization of liver fibrosis in CCl_4_-treated control and BCL6B Tg rats by Sirius Red staining and immunohistochemical staining of α-SMA. **D.** The downregulation of α-SMA protein and mRNA expression in liver tissues of CCl_4_-treated control and BCL6B Tg rats was confirmed by Western blot and quantitative polymerase chain reaction (PCR), respectively. **E.** Correlation analysis for BCL6B and α-SMA mRNA expression levels in liver tissues from 42 patients with chronic liver disease (CLD) using quantitative PCR. *p < 0.05, **p < 0.01.

Liver fibrosis is defined as the accumulation of excessive amounts of extracellular matrix (ECM) in the liver parenchyma [6]. In liver injury models, ectopic expression of BCL6B resulted in significantly lower mRNA levels of collagen type I (Coll-1) and tissue inhibitor of metalloproteinases 1 (TIMP1), a gene related to ECM deposition (Figure 4A). Serum hyaluronic acid (HA) levels, a marker of fibrosis widely used in the clinic, were markedly reduced in BCL6B Tg rats, compared with control rats (Figure 4B). Ectopic expression of BCL6B led to impaired accumulation of collagen in the liver, as measured by Sirius Red staining (Figure 4C). Compared with control rats, decreased α-SMA expression in the liver was also found in CCl_4_-treated BCL6B Tg rats (Figure 4C and 4D). In contrast, neither BCL6B Tg nor control rats showed significant liver fibrosis under the no-treatment conditions (Supplementary Figure 5). Notably, a striking correlation between BCL6B and α-SMA expression was observed in hepatic tissues from 42 patients with CLD (Figure 4E). The above results suggest that BCL6B plays a crucial role not only in liver injury but also in the development of liver fibrosis.

### BCL6B inhibits the activation of HSCs through regulation of HGF

**Figure 5:**
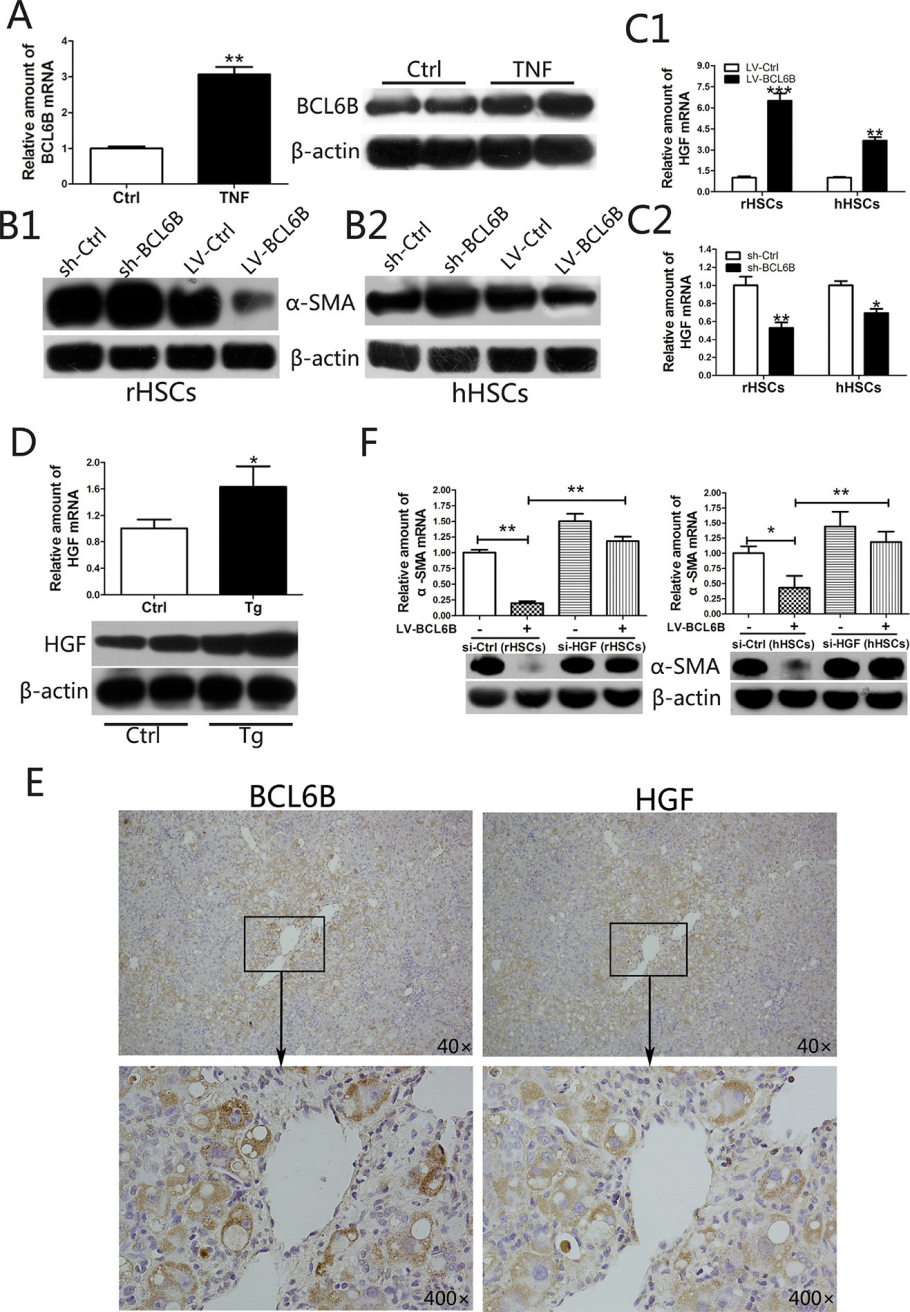
BCL6B inhibits the activation of hepatic stellate cells (HSCs) through regulation of hepatocyte growth factor (HGF) **A.** Expression of BCL6B mRNA and protein in untreated (control) and TNF-stimulated (10 ng/mL for 24 h) HSC-T6 cells (rHSCs). **B.** Analysis of α-SMA protein in B1. rHSCs or B2. LX-2 cells (hHSCs) with or without BCL6B under stimulation by TNF (10 ng/mL for 24 h). **C.** HGF mRNA levels were analyzed after lentivirus-mediated BCL6B overexpression C1. or shRNA-mediated knockdown C2. both in rHSCs and hHSCs under stimulation by TNF (10 ng/mL for 24 h). **D.** Expression of HGF mRNA and protein were analyzed in liver tissues from CCl_4_-treated control and BCL6B Tg rats (*n* = 8). **E.** Immunochemical staining of BCL6B and HGF revealing strong HGF immunosignals in BCL6B-positive cells in BCL6B-Tg rat liver tissues. **F.** siRNA-mediated knockdown of HGF significantly reversed the BCL6B-induced downregulation of α-SMA expression in activated rHSCs (left) and hHSCs (right). *p < 0.05, **p < 0.01, ***p < 0.001.

In an *in vitro* model of HSC activation, we observed higher BCL6B expression levels in response to TNF stimulation in rat HSC-T6 cells (rHSCs) (Figure 5A). The enhanced BCL6B was also observed in activated primary cultured rat HSCs (day 7), compared to quiescent HSCs (day 2; Supplementary Figure 6). To investigate the kinetics of BCL6B-inhibited α-SMA expression, HSCs were cultured in the absence or presence of BCL6B under stimulation by TNF. After knockdown of BCL6B by shRNA, the expression levels of α-SMA were increased in activated rHSCs; on the other hand, lentivirus-mediated overexpression of BCL6B (LV-BCL6B) decreased α-SMA expression levels (Figure 5B1). In activated human LX-2 cells (hHSCs), the expression of α-SMA was altered to a similar extent as that in rHSCs in the absence or presence of BCL6B (Figure 5B2). These results indicate that BCL6B expression is inducible during HSC activation and in response to proinflammatory cytokine stimulation.

It was reported that HGF is a potent anti-fibrotic cytokine that prevents HSC activation and degrades the ECM *in vivo* [21]. HGF can significantly abrogate TGF-β1-induced α-SMA expression in a dose-dependent manner. [22] We found that BCL6B transfection in activated rHSCs or hHSCs led to an increase in HGF mRNA expression levels (Figure 5C1). Meanwhile, knockdown of BCL6B by shRNA significantly decreased HGF mRNA expression levels (Figure 5C2). In addition, increased HGF expression in CCl_4_-treated liver tissues was also observed in BCL6B Tg rats compared with CCl_4_-treated control rats (Figure 5D). Immunohistochemistry analysis revealed a strong HGF immunosignal in BCL6B-positive cells in BCL6B Tg liver tissues (Figure 5E). In contrast, BCL6B Tg and control rats showed similar levels of HGF under the no-treatment conditions (Supplementary Figure 7). In the model of activated primary cultured rat HSCs, enhanced HGF and reduced α-SMA was observed in the BCL6B-Tg cell, compared to control cells (Supplementary Figure 8). To investigate the role of HGF in BCL6B-mediated activation of HSCs, we used a small interfering RNA (siRNA) to knockdown HGF in rHSCs and hHSCs. Knockdown of HGF by siRNA significantly reversed BCL6B-induced downregulation of α-SMA expression in rHSCs and hHSCs under TNF stimulation (Figure 5F). The anti-inflammatory and anti-fibrotic effects of BCL6B were significantly reduced or vanished when HGF was silenced in HSCs (Supplementary Figure 9). These results indicate BCL6B to be an important regulator of HGF in activated HSCs.

### Blocking BCL6B gene expression aggravates liver fibrosis

**Figure 6:**
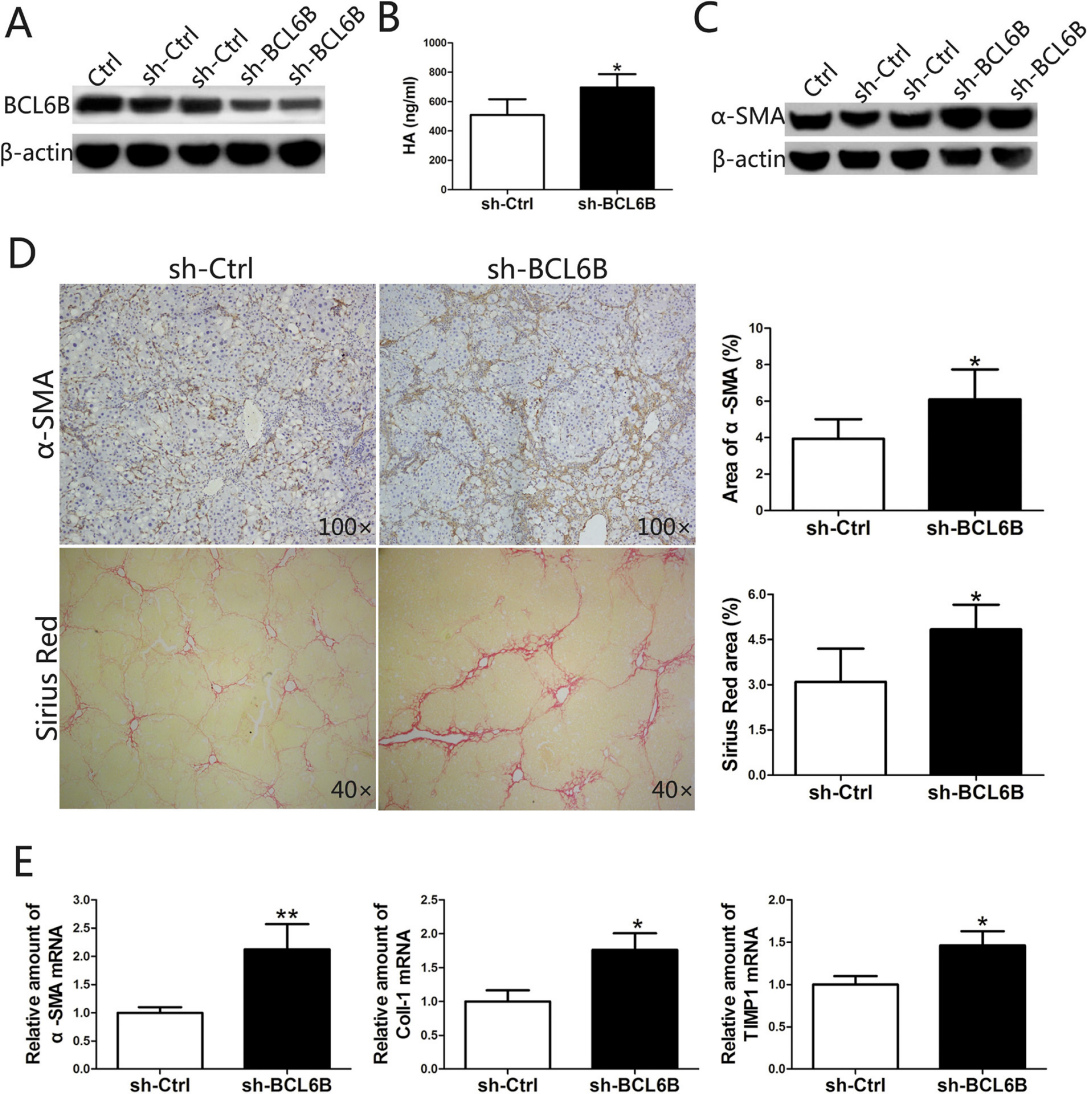
Inhibition of BCL6B expression in the liver aggravates liver fibrosis **A.** Effective knockdown of BCL6B in liver tissue. Rats were injected with PBS (Ctrl), sh-Ctrl or sh-BCL6B adenoviruses via the portal vein, and liver tissues were harvested and analyzed by Western blot after 48 h of DEN treatment. Each lane represents a single rat. **B.** The serum levels of HA were significantly higher in sh-BCL6B-transfected rats compared with sh-Ctrl rats. **C.** Enhanced HSC activation after *in vivo* BCL6B RNA interference. Expression levels of α-SMA and β-actin in liver tissues of Ctrl, sh-Ctrl and sh-BCL6B adenovirus-infected rats were analyzed. Each lane represents a single rat. **D.** Aggravated liver fibrosis upon *in vivo* BCL6B knockdown. Histological characterization of liver fibrosis in sh-Ctrl and sh-BCL6B treated rats (*n* = 8) by α-SMA and Sirius Red staining. Quantitative analysis is shown in the right panel. **E.** Expression of α-SMA, Coll-1 and TIMP1 mRNA in liver tissues of sh-Ctrl and sh-BCL6B adenovirus-infected rats. *p < 0.05, **p < 0.01.

Since BCL6B ameliorated the development of liver fibrosis in CLI rat models, we attempted to promote the progression of liver fibrosis by blocking BCL6B expression in the ALI model. After injecting 2 × 10^10^ copies of the Ad-shRNA genome through the portal vein, the rats were injected i.p. with DEN. This adenovirus-mediated RNA interference approach was confirmed to knockdown the expression of BCL6B in the liver effectively (Figure 6A). Significantly higher levels of HA, a marker of liver fibrosis, were found in the serum of sh-BCL6B-transfected rats (Figure 6B), compared with control (sh-Ctrl) rats. As indicated by enhanced α-SMA protein expression in whole liver lysates (Figure 6C), knockdown of BCL6B promoted the activation of HSCs under the stimulation of DEN. Enhanced α-SMA staining was also observed in the liver tissues of sh-BCL6B-transfected rats (Figure 6D). More importantly, collagen deposition in the liver was enhanced markedly after BCL6B interference (Figure 6D). Similarly, significantly increased mRNA levels of α-SMA, Coll-1 and TIMP1 were found following BCL6B interference (Figure 6E). In contrast, neither sh-BCL6B-transfected nor control rats showed significant liver fibrosis under the no treatment conditions (Supplementary Figure 10). These results demonstrate that the progression of liver fibrosis can be promoted by blocking BCL6B expression *in vivo*.

### The anti-tumor efficacy of BCL6B in HCC and gene expression microarray analysis

The anti-tumor efficacy of BCL6B in HCC, which has been described by Jia Wang *et al.* [18], was confirmed in our study. *In vitro*, overexpression of BCL6B not only inhibited cell proliferation (Supplementary Figure 11), induced apoptosis (Supplementary Figure 12), and inhibited the migration and invasion (Supplementary Figure 13) of HepG2 and SMMC-7721 cells but also impaired cell cycle progression (Supplementary Figure 14), leading to cell cycle arrest. *In vivo*, upregulation of BCL6B by lentiviruses also significantly suppressed the growth of subcutaneous tumors in nude mice (Supplementary Figure 15).

To identify the mechanism by which BCL6B regulates HCC cellular function, the genome-wide transcription patterns of HepG2 cells with or without stable BCL6B expression were compared to identify the pathways that might be altered by BCL6B. A total of 553 differentially expressed genes (fold change ≥ 1.5, *p* < 0.05) were identified in HepG2 cells transfected with LV-BCL6B versus LV-Ctrl. The data were further analyzed using an online database: http://david.abcc.ncifcrf.gov/. The gene ontology analysis and Kyoto Encyclopedia of Genes and Genomes (KEGG) analysis indicated the involvement of the differentially expressed genes and signaling pathways in the regulation of cell cycle, apoptosis, adhesion, and migration (Supplementary Figure 16A and 16B). Several critical genes differentially expressed in BCL6B-expressing cells, with their fold expression changes and gene functions, are detailed in Table 3.

**Table 3:** Stable expression of BCL6B in HepG2 cells promotes pathways that impair cell-cycle progression, activate cell death and inhibit cell migration

Gene	Δ(fold)	Gene function
*Regulators of cell-cycle progression*		
CDT1	1.535	Present during G1 and early S phase of the cell cycle. Degraded during the late S, G2, and M phases
RASSF4	1.619	Potential tumor suppressor. May promote apoptosis and cell cycle arrest.
C13orf15	0.583	Modulates the activity of cell cycle-specific kinases. Enhances CDC2 activity.
CCND2	1.758	Cyclin D2, Essential for the control of the cell cycle at the G1/S (start) transition
CDKN2A	0.599	Capable of inducing cell cycle arrest in G1 and G2 phases
CDKN2B	0.514	Interacts strongly with CDK4 and CDK6. Potential effector of TGF-beta induced cell cycle arrest.
CKAP2	0.599	Accumulates as cells progress from S to G2 into mitosis.
MAP3K8	1.962	Isoform 1 is activated specifically during the S and G2/M phases of the cell cycle.
PPM1D	1.605	Required for the relief of p53-dependent checkpoint mediated cell cycle arrest.
RCC1	1.545	Involved in the regulation of onset of chromosome condensation in the S phase
*Regulators of migration and adhesion*		
CDH1	2.017	E-cadherin, involved in mechanisms regulating cell-cell adhesions, mobility and proliferation of epithelial cells
CYR61	0.507	Promotes cell proliferation, chemotaxis, angiogenesis and cell adhesion
DPP4	2.142	Overexpression in HEK293 cells reduced cell adhesion, migration and invasion on ECM components, increased proliferation and promoted apoptosis
ICAM1	1.601	Intercellular adhesion molecule 1
CEACAM	0.636	Carcinoembryonic antigen-related cell adhesion molecule 1
PLAU	0.490	Possible mediators in HGF-induced migration.
CD36	2.198	May function as a cell adhesion molecule.
*Regulators of apoptosis and cell death*		
BCL3	1.670	Overexpression of BCL3 increases apoptosis
BNIP3	2.815	Apoptosis-inducing protein which can overcome BCL2 suppression
TP53INP1	1.644	Functionally associated with p73 to regulate cell cycle progression and apoptosis
CD24	0.646	CD24 appears to be highly expressed in a large variety of human cancers and to contribute to the acceleration of tumor growth and metastases
PAK2	0.516	Full length PAK 2 stimulates cell survival and cell growth.
PDCD5	2.145	May function in the process of apoptosis. Activated in cells undergoing apoptosis
CTNNB1	0.649	A role for beta-catenin in the control of cell cycle and apoptosis at G2/M.

## DISCUSSION

Liver cancer and cirrhosis represent a major global health concern. In this study, we showed for the first time that BCL6B expression is decreased in HCC tissues compared with non-cancer tissues, and BCL6B expression was correlated with the clinicopathological characteristics of HCC, especially cirrhosis. Reduced BCL6B expression, both at the mRNA and protein levels, in liver tissues was confirmed by quantitative PCR, immunohistochemistry and Western blot. Low expression of BCL6B correlated with shorter survival in HCC patients of the TMA cohort, and multivariate analysis indicated that BCL6B was an independent prognostic indicator of HCC. Furthermore, the BCL6B risk score significantly increased the prognostic value of the TNM stage, histopathologic grading and preoperative AFP level. These findings indicate that BCL6B may be involved in the progression of HCC and be a significant prognostic indicator for HCC patients.

Decreased BCL6B expression was associated with HCC, but high expression was significantly associated with cirrhosis, as confirmed in both the quantitative PCR and TMA cohorts. To test our hypothesis that BCL6B plays a pivotal role in regulating the progression of cirrhosis to HCC, we performed a series of cell and animal experiments to explore the potential responsible mechanisms. In this study, enhanced BCL6B expression was discovered both in ALI and CLI rats treated by DEN or CCl_4_, indicating that BCL6B may promote resistance to liver injury. In this study, the methods of overexpression or knockdown of BCL6B via portal vein injection of lentiviruses or adenoviruses were shown effectively. Overexpression of liver-specific BCL6B by lentiviruses *in vivo* not only ameliorated hepatocellular damage and inflammation but also attenuated the procession of liver fibrosis in the CLI model. Furthermore, inhibiting BCL6B expression by adenovirus-mediated RNA interference aggravated liver fibrosis in the ALI model, as expected. In contrast, under the no treatment conditions, neither LV-BCL6B-transfected nor sh-BCL6B-transfected rats showed any significant differences in inflammatory cell infiltration or liver fibrosis, compared with control liver tissues. It was also found that BCL6B might function as an inhibitor of HSC activation by upregulation of HGF. However, the exactly mechanism by which BCL6B regulates the progression of liver fibrosis needs further investigation, and the above results should be confirmed in BCL6B knockout (BCL6B^−/−^) animals.

The lack of sensitive and reproducible markers for liver fibrosis has been a major limitation not only for research and the clinical management of liver diseases, but also for the development of anti-fibrotic drugs [23]. More than 90% of HCCs worldwide develop from a cirrhotic background, which urged us to identify an effective marker of both HCC and cirrhosis. The results here suggest that BCL6B may be an ideal candidate for predicting the progression of both HCC and liver fibrosis, since BCL6B expression was reduced in HCC tissues but enhanced in fibrotic tissues. The opposing directions of BCL6B expression indicate that BCL6B plays an antifibrogenic role at the cirrhosis stage, but when BCL6B is silenced or loses its normal function, tumorigenesis is induced. In addition, the mRNA levels of BCL6B in HCC tissues were positively correlated with the presence of hepatitis B virus (HBV) in quantitative PCR cohort (*p* < 0.05; Table 1). HBV is a major causative agent of chronic liver disease and subsequent liver cirrhosis worldwide [24]. It was reported that HGF restrains hepatic injury, conferring protection from hepatitis and fibrosis [25]. In this study, it demonstrated that BCL6B may play a pivotal role in the process of hepatitis development through upregulation of HGF, but the exactly mechanism needs further investigation. All the results above provide a basis for improving the diagnosis and therapy of liver damage.

The antitumor efficacy of BCL6B in HCC both *in vitro* and *in vivo* reported by Jia Wang *et al.* [18] was confirmed in this study. We discovered that overexpression of BCL6B impaired cell cycle progression, leading to cell cycle arrest. To understand the anti-tumor mechanisms better, genome-wide transcriptome array analysis was performed to provide a broad overview of the biological function of the genes affected by BCL6B overexpression. Several of the genes found to be differentially expressed are crucial in the development of malignant tumors. The efficacy of inhibiting metastases in HCC, which was also reported by Jia Wang *et al*., was confirmed by the identification of differentially expressed genes such as CDH1, CD36, and ICAM1 in our study. In the analysis of the clinicopathological characteristics, the correlation between reduced BCL6B expression and high frequency PVTT confirmed the above results. As a consequence, BCL6B was confirmed for its anti-tumor efficacy and was identified as a pivotal tumor suppressor gene in HCC.

Taken together, the data presented here showed that the loss of BCL6B expression was significantly correlated with HCC progression and was an independent prognostic marker of poor overall survival in HCC patients, as well as added significant prognostic value to well-known clinical prognostic factors. BCL6B inhibited the activation of HSC though upregulation of HGF, a well-known antifibrogenic mediator, leading to amelioration of hepatocellular damage and liver fibrosis. Based on this study, we propose that BCL6B may serve as a promising prognostic marker for HCC, and restoration of BCL6B expression may be a novel strategy for antifibrogenic therapy for human HCC.

## MATERIALS AND METHODS

### Patients and specimen collection

Patients were recruited from the First Affiliated Hospital of Zhejiang University, Hangzhou, China. The patients were divided into two independent cohorts as follows. The tissue microarray (TMA) cohort included 90 HCC patients who underwent hepatectomy from May 2007 to November 2012 with completed survival data, and the quantitative PCR cohort consisted of 126 surgical cases from December 2008 to April 2011. For liver fibrosis analysis, tissues from 42 patients with chronic liver disease (CLD) were collected. The study was approved by the local Ethics Committee, and informed consent was obtained from all patients.

### TMA construction and immunohistochemistry

HCC TMAs were designed by a contract service at the National Engineering Centre for Biochip, Shanghai, China, and a standard protocol was used for immunostaining of the TMAs, as described in the Supplementary Data.

### Assessment of immunohistochemistry

Staining of BCL6B in tissue samples from the TMA cohort was scored independently by two pathologists blinded to the clinical data, by applying a semi-quantitative immunoreactivity score (IRS), as reported elsewhere [26]. The optimal cut-off value for the IRS was obtained by receiver operating curve (ROC) analysis, as described in the Supplementary Data. Under these conditions, samples with an IRS of 0─4 and 6─12 were classified as low and high BCL6B expression, respectively.

### Quantitative real-time PCR analysis

Total RNA was extracted from cell lines or tissues and used to synthesize cDNA (Bio-Rad, Hercules, California, USA). Real-time PCR was performed according to the manufacturer's instructions. The primer sequences are shown in Supplementary Table 1.

### Construction of a BCL6B expression lentivirus and short hairpin RNA

A replication-defective lentivirus encoding the complete BCL6B open reading frame (LV-BCL6B) and adenovirus-mediated short hairpin RNA specific for BCL6B (sh-BCL6B) were constructed by Invitrogen (Carlsbad, CA, USA). LV-Ctrl and sh-Ctrl were used as the negative controls, respectively. The sh-BCL6B sequence used was 5′-GCGCACAAGGCAGTTCTTATT-3′.

### Preparation of cell lines and animals

HSC-T6 rat hepatic stellate cells and LX-2 human hepatic stellate cells were grown in Dulbecco's Modified Eagle's Medium (DMEM), supplemented with 10% fetal bovine serum (FBS) and 100 U/ml penicillin/streptomycin. Other cell culture methods are detailed in the Supplementary Data.

All animal experiments were conducted in accordance with *The Guide for the Care and Use of Laboratory Animals at Zhejiang University*. Male Sprague–Dawley rats, weighing 180–220g, were used in this study. On the day of surgery, the rats were anesthetized with chloral hydrate. After a midline laparotomy, lentivirus or adenovirus in 2-ml phosphate-buffered saline (PBS) were injected through the portal vein, and the abdomen was closed. The concentration of LV-BCL6B or LV-Ctrl was 5 × 10^7^ Tu/ml, and the concentration of adenovirus-mediated sh-BCL6B or sh-Ctrl was 1 × 10^9^ Tu/ml. Fourteen days after surgery, the rats were used to establish the liver fibrosis models.

### Models of liver fibrosis

For the acute liver injury (ALI) models, rats were treated with 100 μg/g body weight diethylnitrosamine (DEN; Sigma-Aldrich, St. Louis, MO, USA) in PBS (15 ml/g body weight) by intraperitoneal (i.p.) injection. Rats from the control group were injected with PBS only. Rats were killed 48 h after DEN treatment.

For the chronic liver injury (CLI) models, rats were treated with 2 μl/g body weight CCl_4_ (Sigma-Aldrich), diluted 4× in corn oil (Sigma-Aldrich), by i.p. injection twice weekly for 8 weeks. Rats from the control group were injected with corn oil only. Rats were sacrificed and the tissues harvested 48 h after the final CCl_4_ injection.

### Quantitative and statistical analyses

Collagen distribution was analyzed using the Image-Pro Plus software (Media Cybernetics, Bethesda, MD, USA) to quantify the positively stained tissue area. Data are displayed as means ± standard deviations. The associations between BCL6B expression and clinicopathological parameters were evaluated by Fisher's exact test. Differences in the IRS for BCL6B staining between primary tumors and their corresponding non-tumor tissues were assessed by the Wilcoxon test (grouped). The probability of differences in overall survival (OS) was determined by the Kaplan-Meier method, together with the log-rank test for evaluation of significance. Univariate or multivariate Cox regression analysis was used to estimate the hazard ratios (HRs) and their 95% confidence intervals (CIs). ANOVA and Student's *t* test were used to determine the statistical significance of differences between experimental groups *in vitro* and *in vivo*. All statistical analyses were performed using the SPSS 19.0 software for Windows (SPSS, Chicago, IL, USA). *p* < 0.05 was considered to indicate statistical significance. Graphs were created using GraphPad Prism 5.

### Additional methods

The detailed methodology is described in the Materials and Methods section of the Supplementary Information.

## SUPPLEMENTARY MATERIALS AND METHODS


