# Supplementary material for: BCL6B expression in hepatocellular carcinoma and its efficacy in the inhibition of liver damage and fibrogenesis
Source: Oncotarget. 2015 Apr 27;6(24):20252–65. doi: 10.18632/oncotarget.3857 (PMC4653002; doi:10.18632/oncotarget.3857)
Supplement: Supplementary file 1 [file oncotarget-06-20252-s001.pdf]

SUPPLEMENTARY MATERIALS AND METHODS

Cell lines and cell culture

Seven HCC cell lines (HepG2, Hep3B, HuH7, HCCLM3, SK-Hep1, SMMC-7721, Bel-7402), the normal liver cell line L-02, the rat hepatic stellate cell line HSC-T6 and the human hepatic stellate cell line LX-2 were used in this study. Most of the cell lines (HepG2, Hep3B, HUH-7, HCCLM3, SK-Hep-1, HSC-T6 and LX-2) were cultured in Dulbecco's Modified Eagle's Medium (DMEM; Gibco, Grand land, NY, USA), and the remaining cell lines (SMMC-7721, Bel-7402, L-02) were cultured in RPMI-1640 medium (Gibco). All media were supplemented with 10% heat-inactivated fetal bovine serum (FBS) (Sigma–Aldrich, St. Louis, MO, USA) and 100 U/ml penicillin/streptomycin. All cells were maintained in a humidified atmosphere containing 5% CO_2 at 37ºC and were passaged using standard cell culture techniques.

Western blot analysis

Proteins were extracted from tissues and cell lines. The protein concentration was measured using the BCA protein assay (Thermo Scientific, Waltham, MA, USA). Approximately 50 µg tissue or 30 µg cellular protein lysates were separated by electrophoresis using 12% PAGE gels (Invitrogen) and then transferred to polyvinylidene fluoride (PVDF) membranes. After blocking nonspecific binding sites for 2 h with 5% non-fat milk, the membranes were incubated overnight on ice with primary antibodies against BCL6B (ab87228, Abcam, Cambridge, UK) and β-actin (Sigma–Aldrich). After washing the membranes several times in Tris-buffered saline and Tween 20 (TBST) with agitation, detection was performed using the appropriate secondary horseradish peroxidase-linked anti-mouse or anti-rabbit antibody. Membrane-enhanced chemiluminescence was then conducted using a chemiluminescence detection kit (Biological Industries, Beit Haemek, Israel).

Tissue microarray construction and immunohistochemistry

For the construction of TMAs, duplicate 1.0-mm diameter cores of tissue from each sample were punched from paraffin tumor blocks and corresponding non-tumor tissues.

Immunohistochemical analysis was performed using paraffin-embedded tissues. Briefly, serial 4-µm sections were deparaffinized and rehydrated and then subjected to heat-induced epitope retrieval in a microwave oven. Next, the sections were treated with 3% hydrogen peroxide to quench endogenous peroxidase activity, followed by incubation with 5% FBS to block non-specific binding. After blocking, the sections were incubated overnight at 4°C with primary antibodies against BCL6B (ab87228, Abcam), α-SMA (ab5694, Abcam) or CD3 (ab5690, Abcam). The following day, the sections were incubated with HRP-conjugated secondary antibodies (Invitrogen) and visualized using 3, 3′-diaminobenzidine (Zhongshan Golden Bridge Biotechnology, Beijing, China), followed by counterstaining with hematoxylin. For the negative control, the primary antibody was replaced with phosphate-buffered saline (PBS). The staining scores of the tissue controls on each microarray slide were pre-evaluated as a quality control for immunostaining.

Assessment of immunohistochemistry

Tissue staining of BLC6B was scored independently by two pathologists blinded to the clinical data, by applying a semiquantitative immunoreactivity score (IRS), as reported elsewhere (1). Category A comprised the immunostaining intensity, scored as 0–3 (0, negative immunostaining; 1, weak immunostaining; 2, moderate immunostaining; 3, strong immunostaining). Category B comprised the percentage of immunoreactive cells, scored as 1 (0–25%), 2 (26–50%), 3 (51–75%), or 4 (76–100%). Multiplication of category A and B resulted in an IRS ranging from 0 to 12 for each tumor or peritumor. The few discrepancies between the two pathologists regarding the BCL6B IRS were resolved by consensus using a multi-head microscope.

The optimum cutoff value for the IRS was obtained by receiver operating curve (ROC) analysis, and the area under the curve (AUC) values for the various BCL6B IRS cutoff values for 1-, 3-, 5- and 7-year overall survivals were calculated. The optimum cutoff value for the BCL6B IRS was 4, considering that the predictive value of this cutoff point for death was the best (data not shown). Under these conditions, samples with IRS of 0–4 and 6–12 were classified as low and high BCL6B expression, respectively.

Isolation of liver HSCs

Briefly, cells were isolated from the livers using a two-stage collagenase perfusion technique as described previously(2). Filtered cells were centrifuged at 50 g for 2 min to remove hepatocytes. The remaining non-parenchymal cell (NPC) fraction was collected. For HSC enrichment, the remaining NPC fraction was resuspended in 11.5% OptiPrep (Axis-Shield, Oslo, Norway) and put between a bottom cushion of 15% OptiPrep and a top layer of PBS. After centrifugation at 1500 g for 15 min, we obtained the HSC fraction at the interface between the top and intermediate layer. The purity of the HSCs fraction

was estimated based on autofluorescence. Cell viability was examined by Trypan blue exclusion. Both cell purity and viability were in excess of 90%.

Serological analysis

Serum liver enzyme concentrations were quantified using the Advia 1800 analyzer (Siemens Healthcare Diagnostics, Eschborn, Germany) and kits from Bayer HealthCare (Leverkusen, Germany).

Analysis of inflammatory cell recruitment

The number of inflammatory cells in the paraffin-embedded liver sections was determined via manual counting of CD3+ cells or hematoxylin-eosin staining. A minimum of five different fields in the evaluated liver sections from three independent wild-type or BCL6B Tg animals were analyzed for CD3+ cells.

Analysis of apoptosis in liver tissues

The livers were isolated, fixed and embedded in paraffin. Apoptosis was determined by counting TdT-mediated dUTP nick-end labeling (TUNEL)-positive cells using the ApoAlert® DNA Fragmentation Assay Kit (Clontech) in accordance with the manufacturer's instructions. A minimum of five different fields in each liver section was used to count the number of signal-positive cells.

Quantification of hepatic cytokine levels

Liver tissue extracts were obtained by homogenization of snap-frozen tissues in Cell Lysis Buffer (Cell Signaling, Danvers, MA, USA) supplemented with 1 mM PMSF and a protease inhibitor cocktail (cOmplete Mini Protease Inhibitor Cocktail Tablets from Roche Diagnostics, Mannheim, Germany) using a homogenizer, followed by sonication using a Sonopuls HD 70 (Bandelin Electronics, Berlin, Germany) and centrifugation. TNF, IL-6 and IL-1 levels in liver tissue homogenates were assessed by sandwich enzyme-linked immunosorbent assay (ELISA) using the rat TNF, IL-6 and IL-1 DuoSet ELISA development kits (R&D Systems Inc., Minneapolis, MN, USA) following the manufacturer's instructions.

siRNA transfection

The double-stranded HGF siRNA duplex and control siRNA duplex were purchased from Invitrogen. For transfection, 60% confluent cells were plated in six-well plates and incubated overnight; 30 pmol HGF siRNA or control siRNA were transfected into the cells using RNAiMax transfection reagent (Invitrogen) according to the manufacturer's instructions. Cells were collected 48 h after transfection.

Lentiviral infection and stable transfection

Replication-defective lentiviruses encoding the complete BCL6B open reading frame (LV-BCL6B) and a lentivirus vector encoding the green fluorescent protein (LV-Ctrl), as a control, were constructed by Invitrogen. HepG2 and SMMC-7721 cells were seeded at a density of 3×10^5 cells/ml in six-well plates for synchronization. After culturing overnight, the cells were treated with lentivirus at a multiplicity of infection (MOI) of 20 pfu per cell in 2% FBS medium with 8 μg/ml Polybrene (Sigma–Aldrich). After infection for 6 h, the medium with the lentiviruses was discarded, and the cells were refreshed with complete growth medium. Infection was efficient, showing almost 90% GFP-positive cells as early as 48 h after infection. Blasticidin S HCl (5 μg/ml) (Invitrogen) was added to the medium to select for stable transformants; blasticidin-resistant colonies were then expanded. The BCL6B-positive colonies were confirmed by Western blot. The stably transfected cells were used to demonstrate the function of BCL6B in HCC cells.

Cell viability assay

Cell viability assay was performed using the Cell Counting kit-8 (Dojindo Laboratories). The cells were plated in 96-well plates at a density of 3×10^3 per well. We added 10 μl of the Cell Counting kit-8 solution to 90-μl growth medium in each well after 24, 48, 72 or 96 h. One hour later, the absorbance at 450 nm was read to determine the cell viability in each well. All experiments were completed in triplicate.

Colony formation assay

For colony formation assays, the LV-CTRL or LV-BCL6B cells were plated (3, 000/dish) in 10-cm dishes. The surviving colonies (> 50 cells) were stained with crystal violet and counted after 10 days of culture. The experiments were performed in triplicate.

Annexin V apoptosis assay

Cells undergoing early and late apoptosis were quantified using a flow cytometer (CYTOMICS FC 500; Beckman Coulter, Miami, FL, USA), following staining with Annexin V-APC and propidium iodide (Sungene Biotech Co., Ltd, Tianjin, China). The assay was repeated three times.

Flow cytometry analysis of cell cycle distribution

Cells used for the cell cycle assays were fixed in 70% ethanol, stored at 4°C for more than 24 h, and stained with DNA PREP (Beckman Coulter) before being subjected to flow cytometry. The experiments were performed in triplicate.

Analysis of cell invasion and migration

Cell invasion and migration assays were performed using 24-well transwell plates (Millipore, Billerica, MA, USA). Filters used for invasion assays were coated with Matrigel (BD Biosciences, San Jose, CA, USA) in the upper compartment before cell seeding. Then, 4×10^4 cells were seeded in the filters, and the lower compartment was filled with cell culture medium supplemented with 20% FBS. The invading or migrating cells on the bottom surface were stained with crystal violet and counted after 24 h of culture and then photographed using a digital microscope. The experiments were performed in triplicate, and three fields were counted per filter in each group.

In vivo animal experiments

Male BALB/c nude mice at 4–5 weeks obtained from Shanghai SLAC Laboratory Animal Co. Ltd. (Shanghai, China) were used in all experiments and kept in laminar flow cabinets under specific pathogen-free conditions. The experiments were performed according to the institutional ethics guidelines. Mice were divided randomly into two groups of 10 mice each. The HCC stably transfected cells were injected subcutaneously into the armpits of mice in a total volume of 100 µl (2×10^6 cells in PBS). Approximately 10 days after cell inoculation, the tumor volumes in each mouse were monitored every 2 days by measuring the length and width using a caliper. The volume was calculated as the length × $(\text{width})^2/2$. Mice were then sacrificed and the tumors removed and weighed.

REFERENCES

1. Wang S, Wu X, Zhang J, Chen Y, Xu J, Xia X, et al. CHIP functions as a novel suppressor of tumour angiogenesis with prognostic significance in human gastric cancer. Gut. 2013; 62:496–508.
2. Chen L, Li J, Zhang J, Dai C, Liu X, Wang J, et al. S100A4 promotes liver fibrosis via activation of hepatic stellate cells. J Hepatol. 2015; 62:156–64.

SUPPLEMENTARY FIGURES AND TABLES

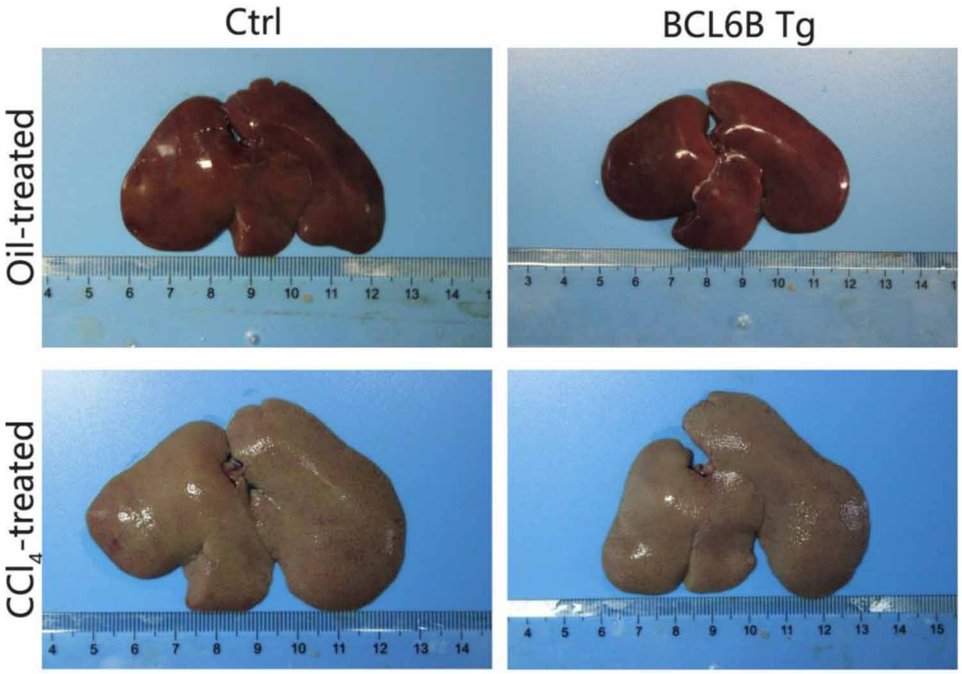

Supplementary Figure 1: The liver morphology of control (Ctrl) and BCL6B transgenic (Tg) rats was similar after the treatment with or without CCl_4 for 8 weeks.

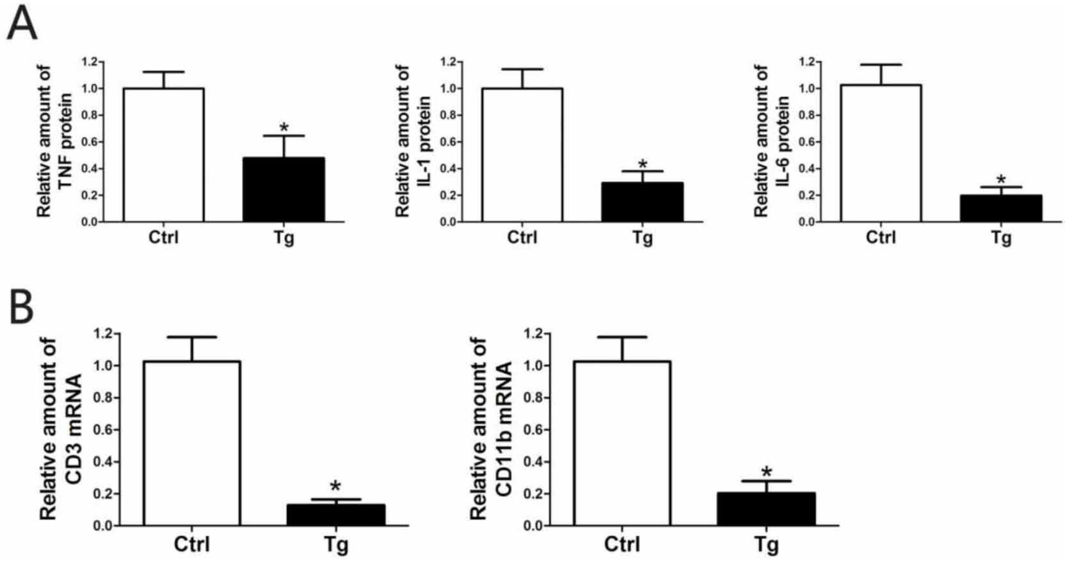

Supplementary Figure 2: Reduced levels of the proinflammatory genes in BCL6B transgenic (Tg) rats compared to control rats under the CCl_4 treatment for 8 weeks. **A.** Quantification of TNF, IL-1 and IL-6 protein levels in liver tissue homogenates by enzyme-linked immunosorbent assay (ELISA). **B.** Analysis of hepatic CD3 and CD11b mRNA expression by quantitative PCR. $*p < 0.01$.

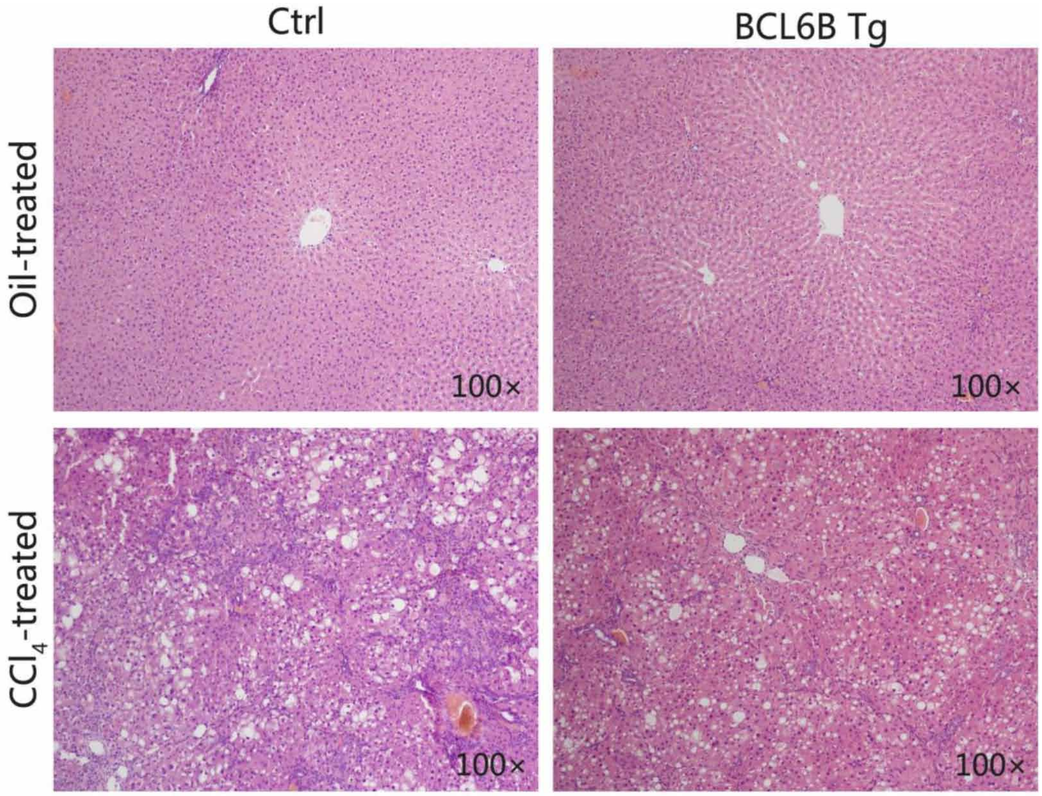

Supplementary Figure 3: Hematoxylin and eosin staining of hepatic tissue from control (Ctrl) and BCL6B transgenic (Tg) rats after the treatment with or without CCl_4 for 8 weeks. The number of recruited inflammatory cells and the area of necrosis were both significantly decreased in the BCL6B Tg rats, compared to control rats under CCl_4 treatment.

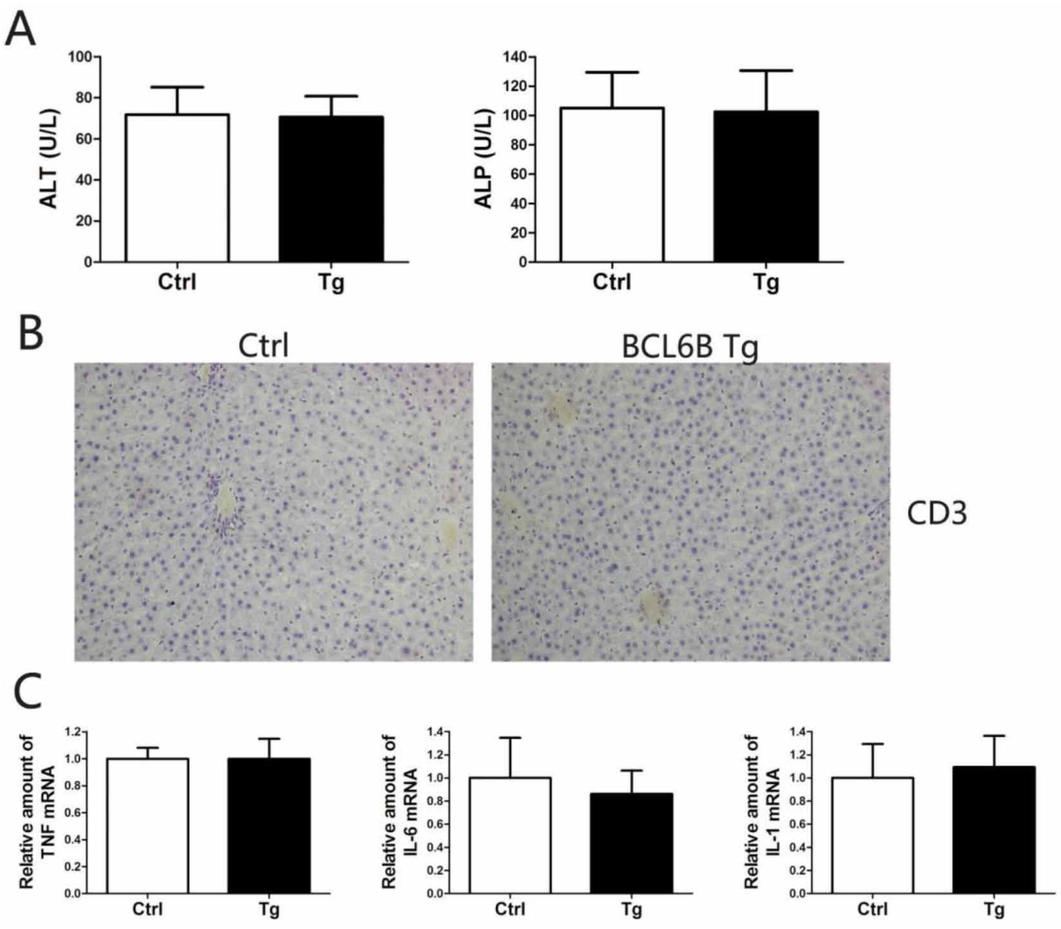

Supplementary Figure 4: A. Quantitative of alanine aminotransferase (ALT) and alkaline phosphatase (ALP) serum levels in no-treatment control (Ctrl) and BCL6B transgenic (Tg) rats. B. CD3 positive immunohistochemical staining of hepatic tissues from no-treatment control and BCL6B Tg rats. **C.** Hepatic mRNA expression of TNF, IL-6 and IL-1 in no-treatment control and BCL6B Tg rats.

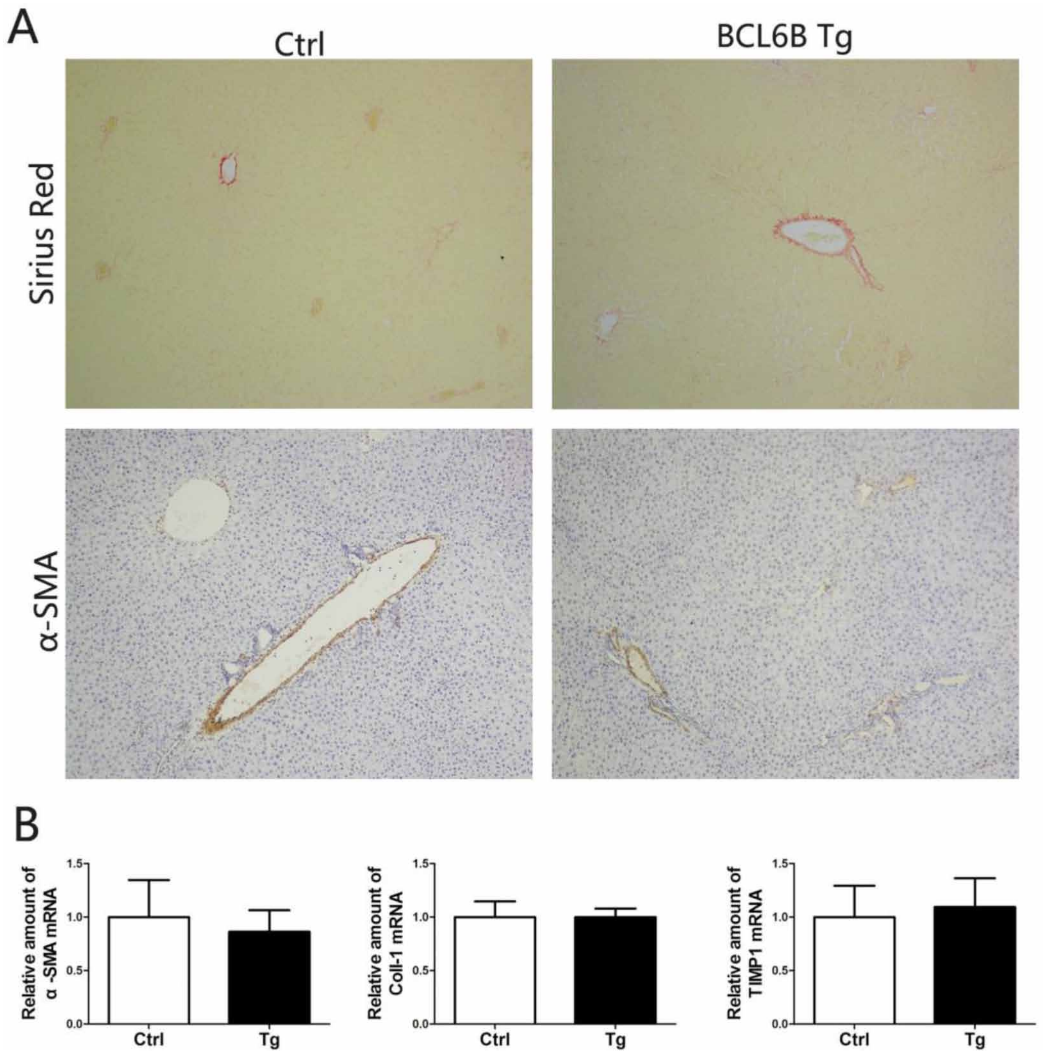

Supplementary Figure 5: Sprague–Dawley rats (control) and BCL6B Tg rats (*n* = 8) were treated with corn oil for 8 weeks. A. Histological characterization of liver fibrosis in oil-treated control and BCL6B Tg rats by Sirius Red staining and immunohistochemical staining of α-SMA. **B.** Expression of α-SMA, Coll-1 and TIMP1 mRNA in liver tissues of oil-treated control and BCL6B Tg rats.

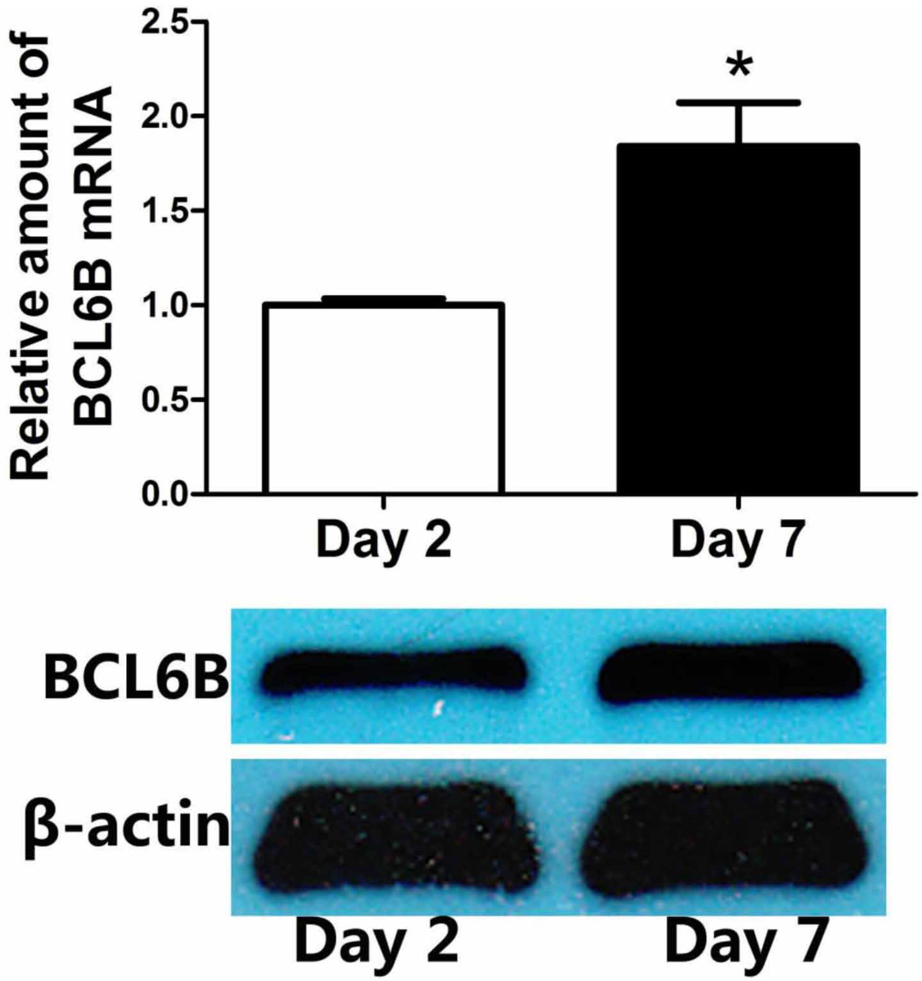

Supplementary Figure 6: Comparison of BCL6B mRNA and protein expression in quiescent (day 2) and activated (day 7) primary cultured rat HSCs using quantitative polymerase chain reaction and western blotting analyses. $*p < 0.01$.

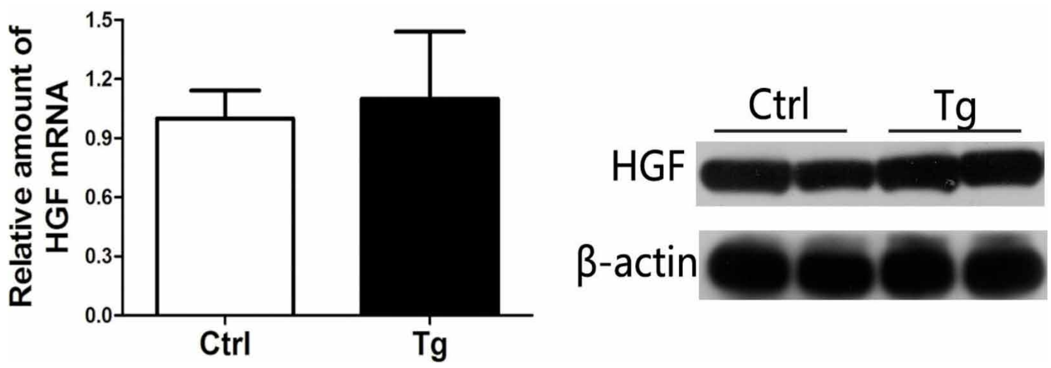

Supplementary Figure 7: Expression of HGF mRNA and protein were analyzed in liver tissues from no-treatment control and BCL6B Tg rats (*n* = 8).

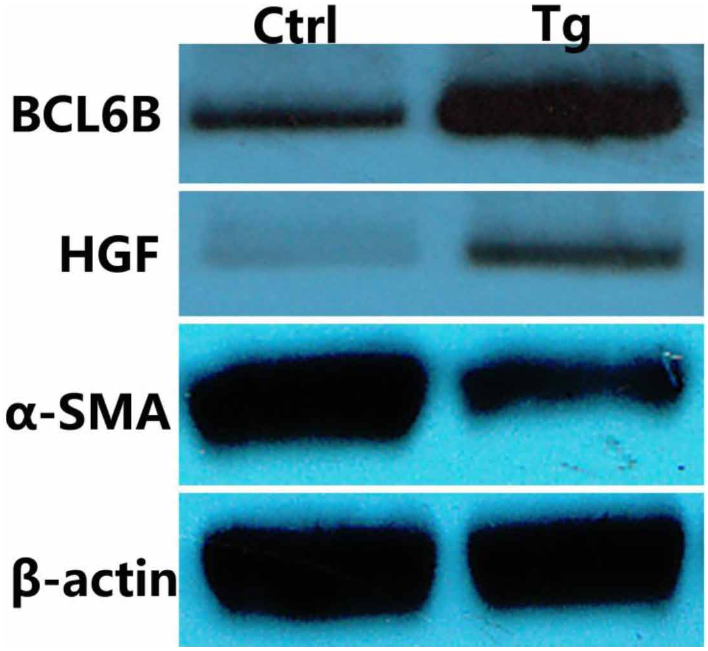

Supplementary Figure 8: Expression of HGF and α-SMA protein were analyzed in the activated primary cultured rat HSCs from control and BCL6B Tg rats.

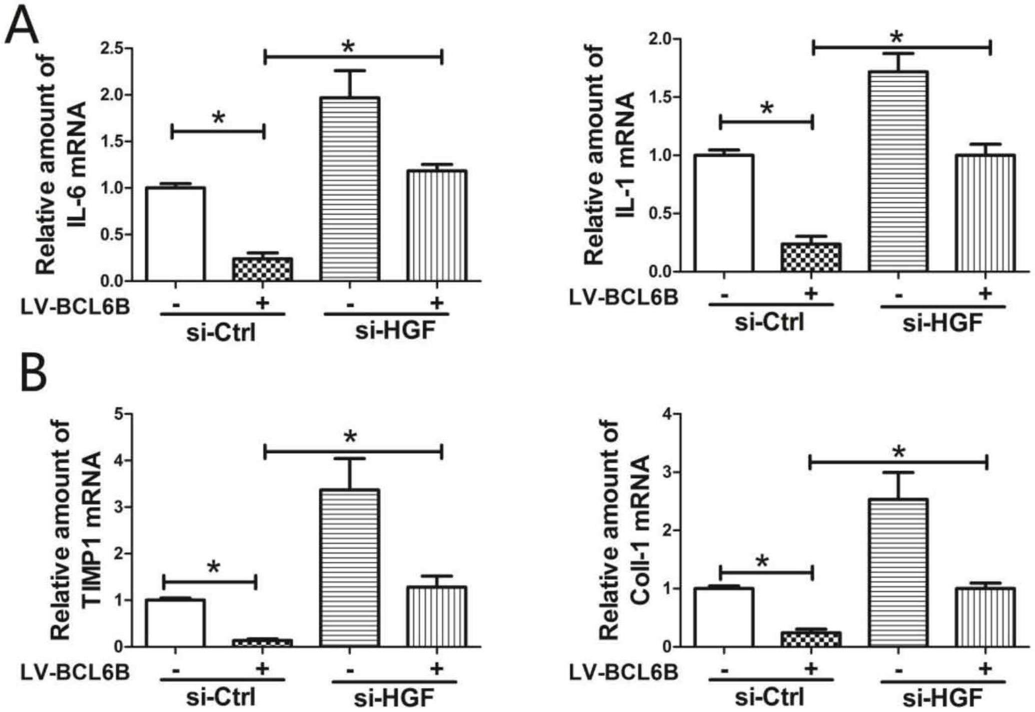

Supplementary Figure 9: The A. anti-inflammatory and B. anti-fibrotic effects of BCL6B were downregulated when HGF was knockdown by siRNA. *p < 0.05.

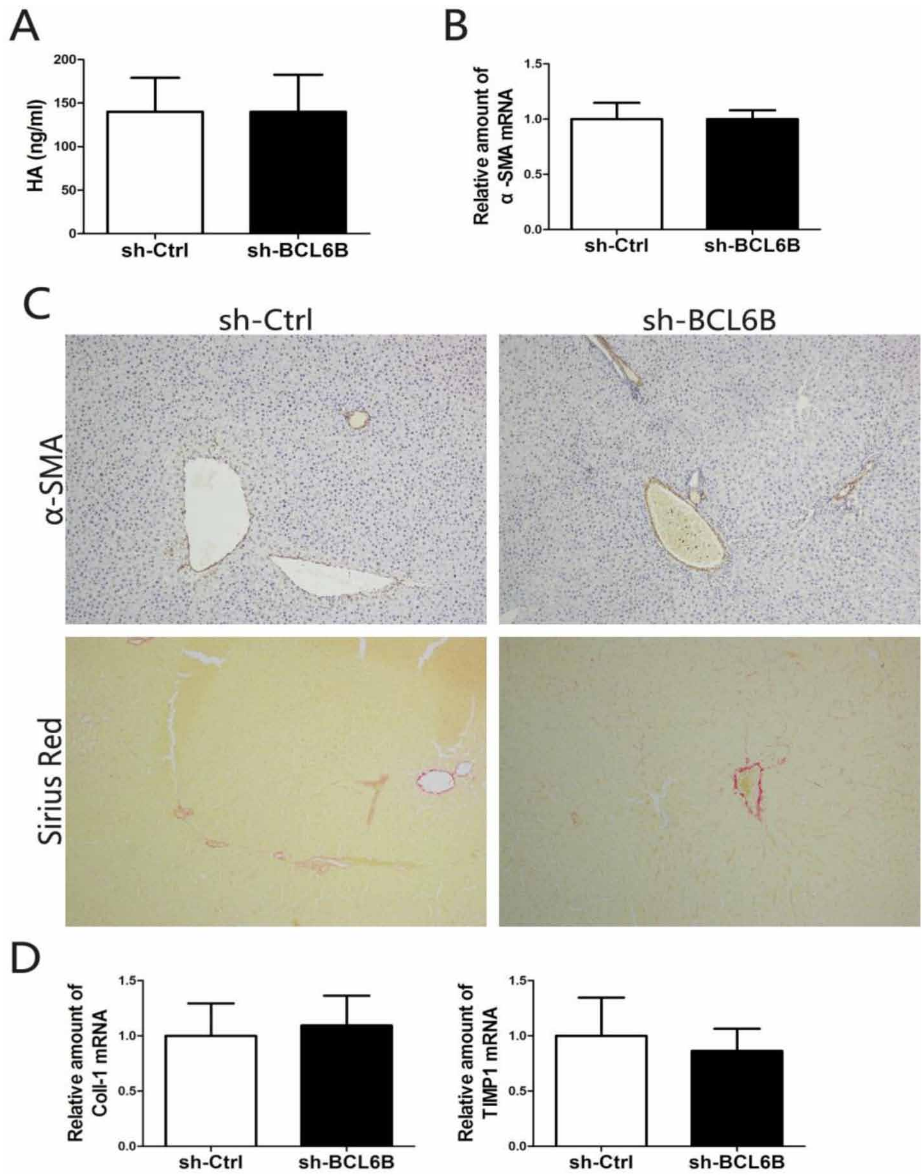

Supplementary Figure 10: Sprague–Dawley rats with negative control sh-RNA (sh-Ctrl) and sh-BCL6B (*n* = 8) were treated with PBS for 48 hours. A. The serum levels of HA of sh-Ctrl-transfected and sh-BCL6B-trasfected rats were shown. **B.** Expression of α-SMA in liver tissues of PBS-treated sh-Ctrl and sh-BCL6B rats. **C.** Histological characterization of liver fibrosis in PBS-treated sh-Ctrl and sh-BCL6B rats by immunohistochemical staining of α-SMA and Sirius Red staining. **D.** Expression of Coll-1 and TIMP-1 in liver tissues of PBS-treated sh-Ctrl and sh-BCL6B rats.

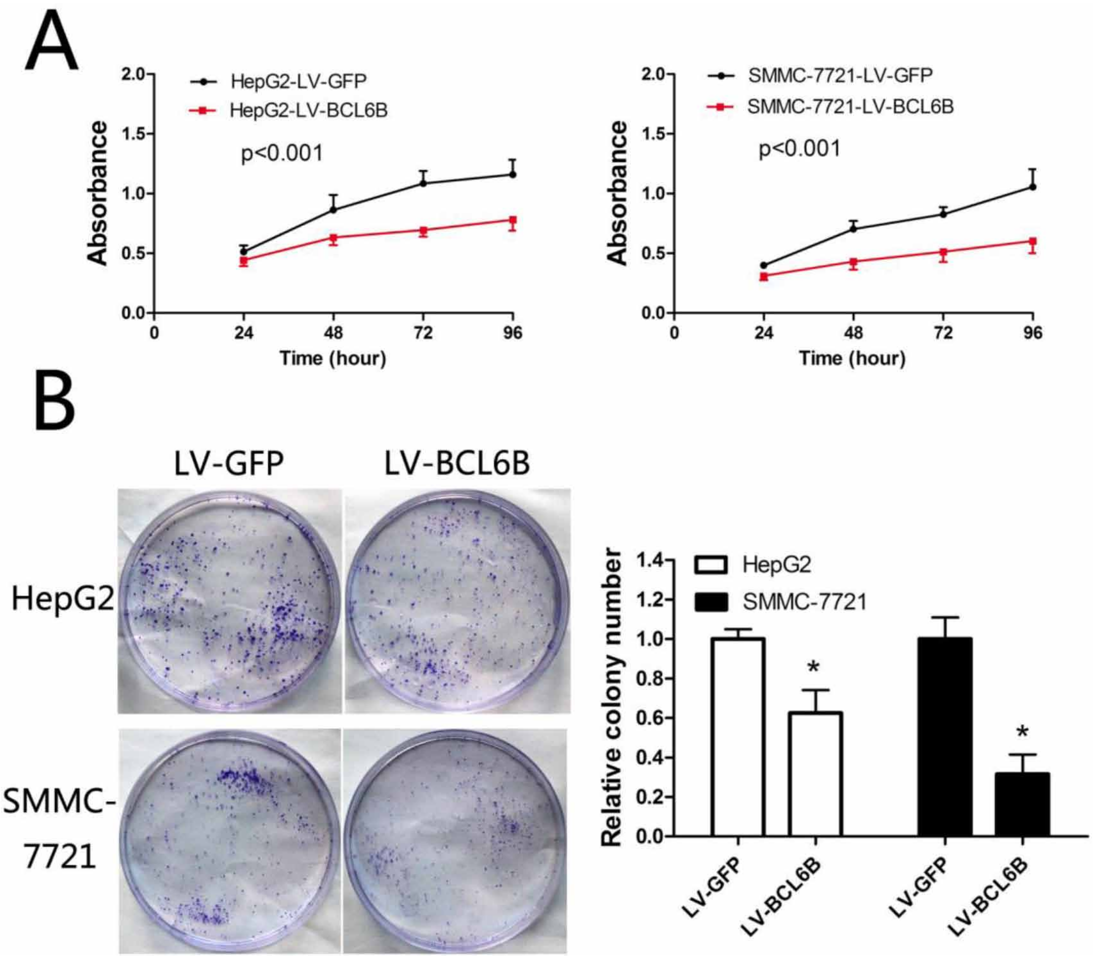

Supplementary Figure 11: Effect of BCL6B expression on tumor proliferation. A. BCL6B significantly inhibited viability of HepG2 ($P < 0.001$) and SMMC-7721 cells ($P < 0.001$). **B.** The effect of BCL6B on cancer cell growth was confirmed by colony formation assay. The left panel shows representative images of colony formation by hepatocellular carcinoma (HCC) cells with stable transfection. Quantitative analysis of colony numbers is shown in the right panel. *$p < 0.01$.

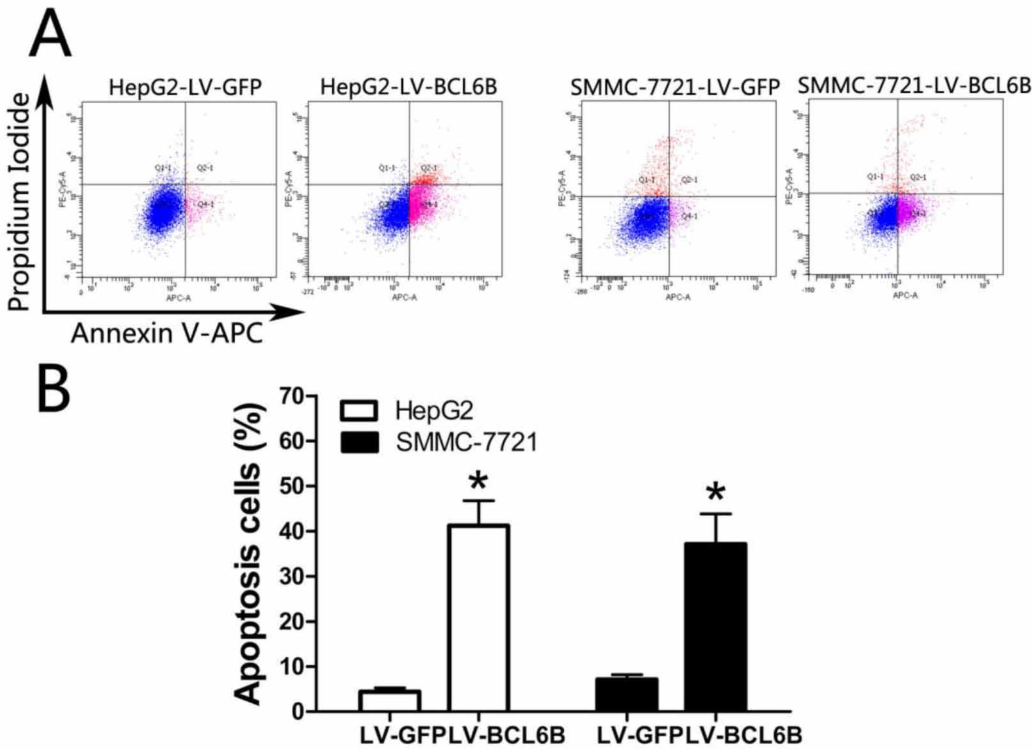

Supplementary Figure 12: BCL6B induced apoptosis in HepG2 and SMMC-7721 cells as shown by flow cytometry following Annexin V-APC and propidium iodide double staining. A. Representative fluorescence-activated cell sorting (FACS) images of HCC cells with LV-GFP or LV-BCL6B. **B.** Quantitative analyses of apoptotic cells (Q2+Q4). The experiment was repeated in triplicate. *$p < 0.001$.

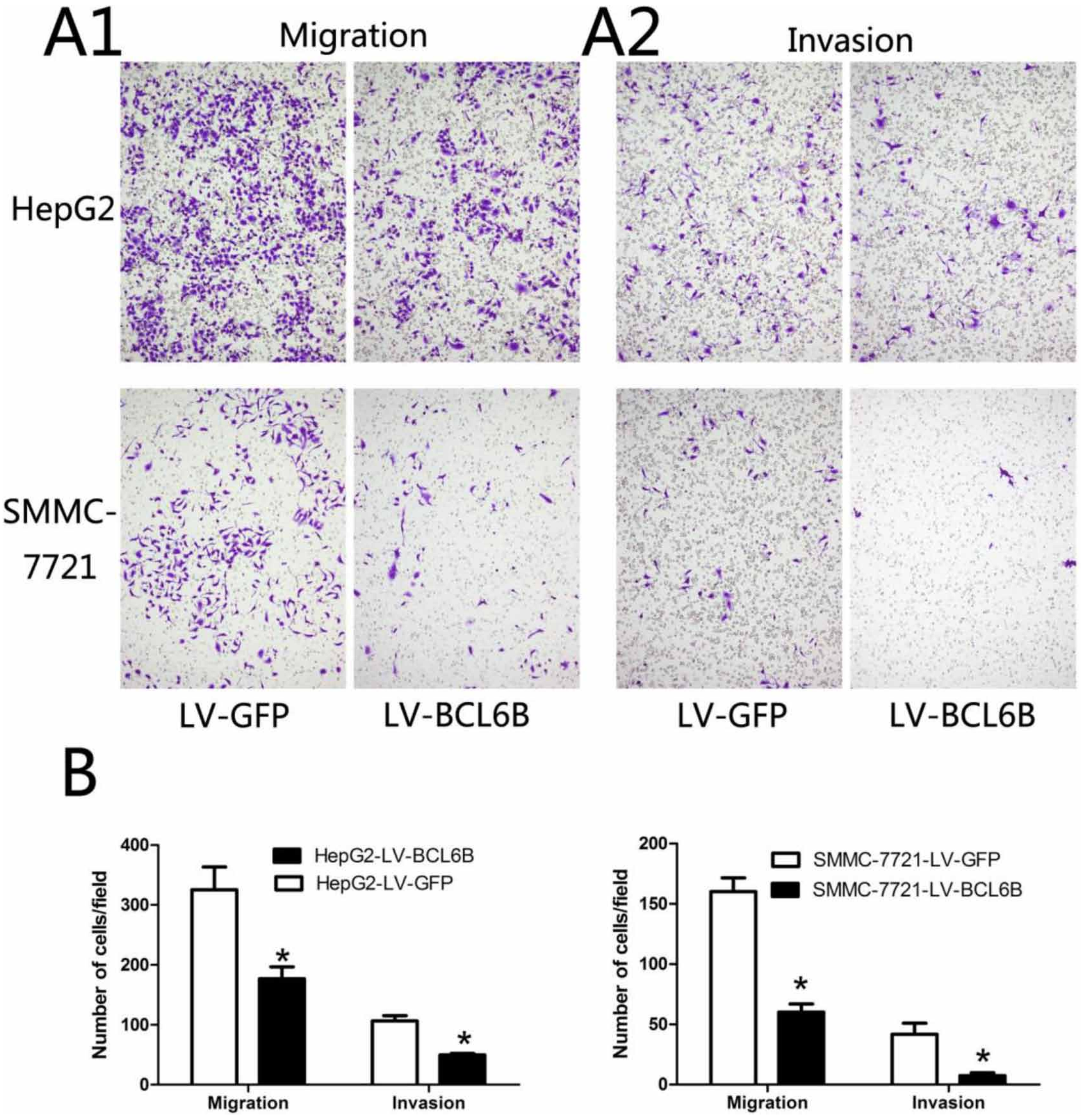

Supplementary Figure 13: BCL6B induced alteration of migration and invasion in hepatocellular carcinoma (HCC) cell lines. A. Representative images of migration **A1.** and invasion **A2.** of HepG2 cells and SMMC-7721 cells using the Matrigel model (original magnification, × 100). **B.** The alteration of migration and invasion was analyzed. BCL6B had an inhibitory effect on migration and invasion. The experiment was repeated in triplicate. *$p < 0.01$.

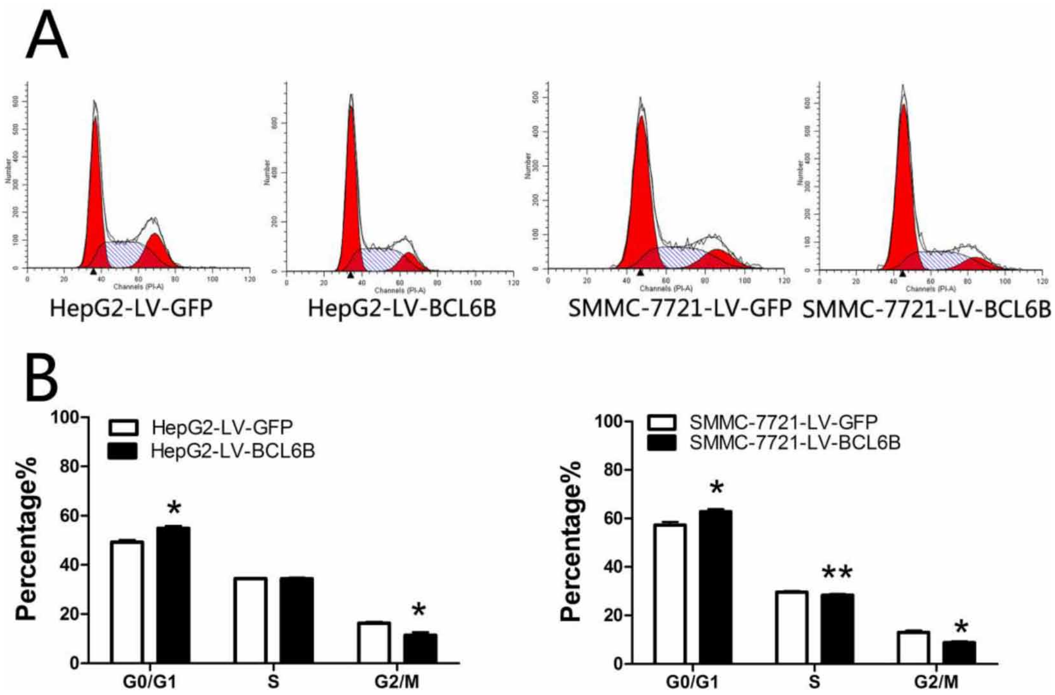

Supplementary Figure 14: BCL6B regulated the cell cycle in HepG2 and SMMC-7721 cells, as shown by cytometry. **A.** Representative FACS images of HCC cells with LV-GFP or LV-BCL6B. **B.** The alteration of cell cycle distribution was analyzed. The experiment was repeated in triplicate. *$p < 0.01$, **$p < 0.05$.

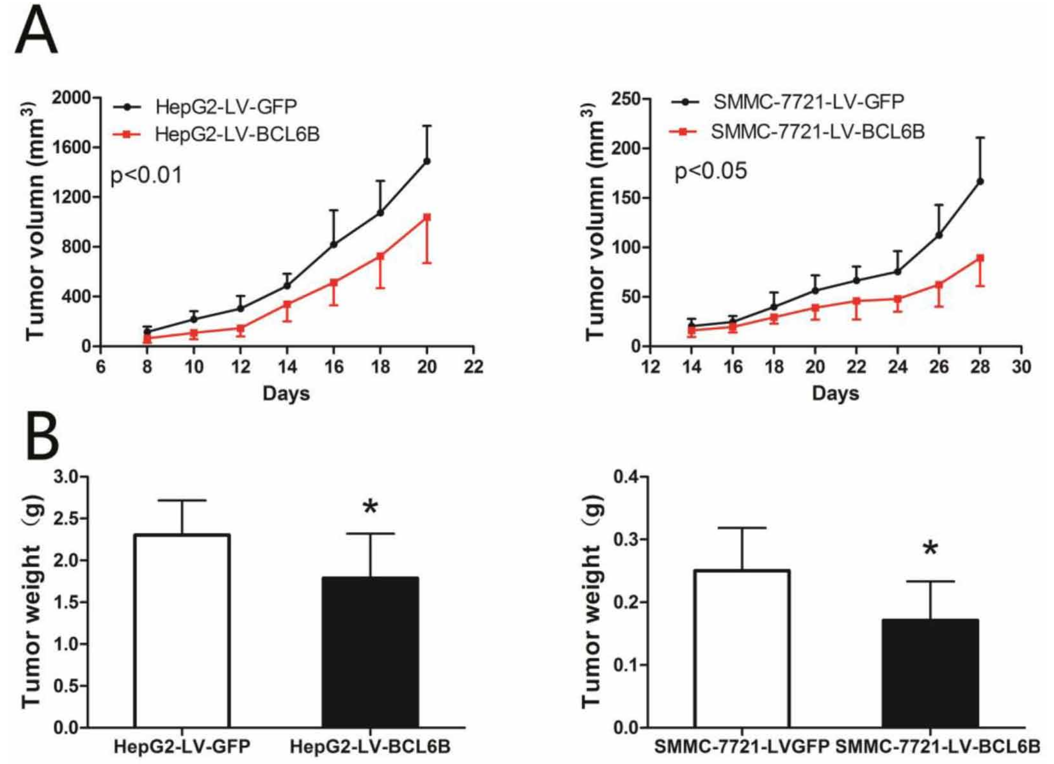

Supplementary Figure 15: BCL6B inhibited growth of tumors derived from HepG2 and SMMC-7721 *in vivo*. **A.** Tumor growth curve of LV-BCL6B cells in nude mice compared with LV-GFP cells ($n = 10$/group). **B.** Histogram showing the mean tumor weights of the LV-BCL6B and LV-GFP groups. *$p < 0.01$.

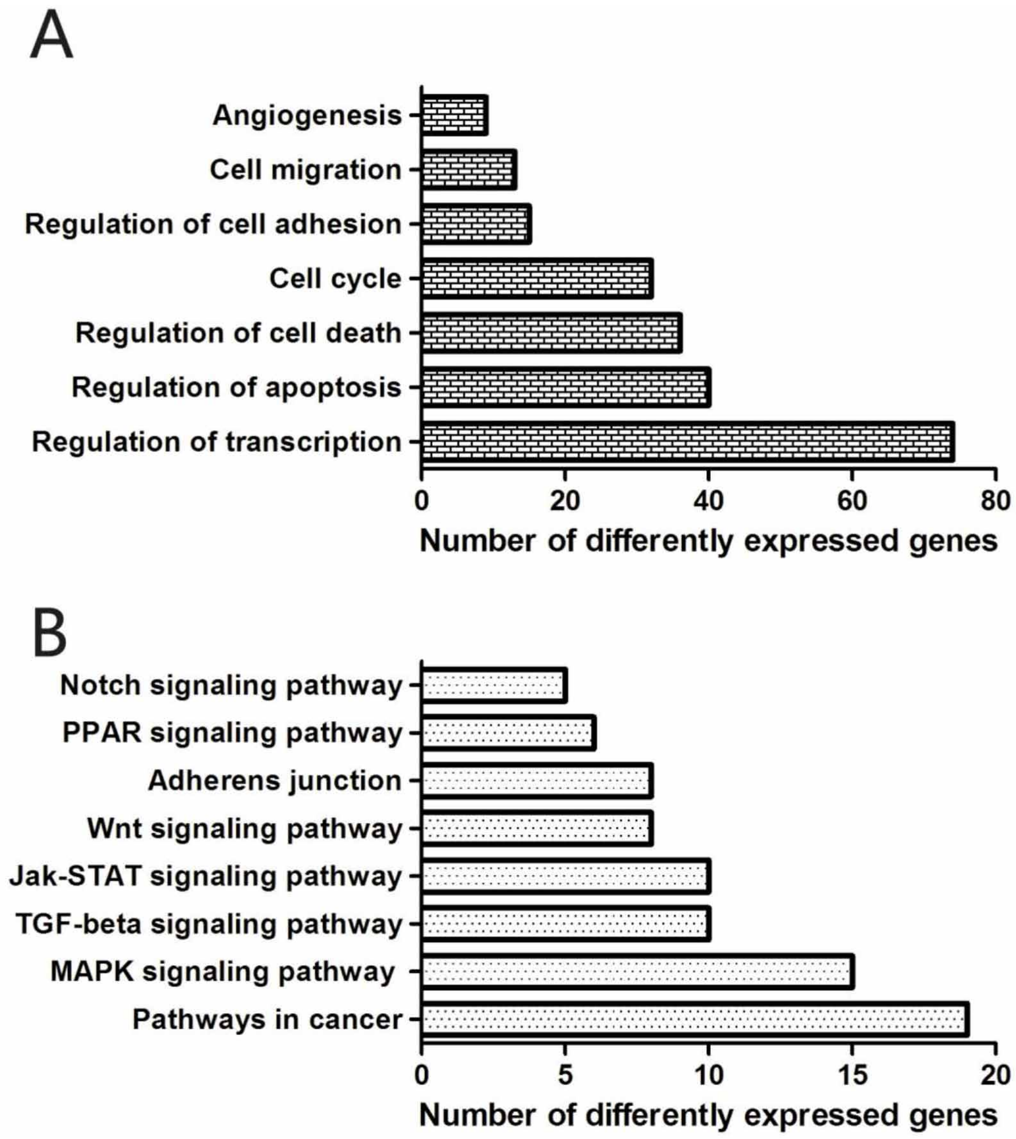

Supplementary Figure 16: A. and B. The gene ontology and KEGG analyses of differentially expressed genes identified by microarray.

Supplementary Table 1: Primers for quantitative real-time PCR

Primer		Sequence (5′-3′)	PCR product (bp)
rBCL6B	Sense	CACTCCTCCGACGTGCTTAG	108
	Antisense	AACTGCCTTGTGCGCTCTTA	
rGAPDH	Sense	GAAACCTGCCAAGTATGATGACA	148
	Antisense	AGCCCCAGCATCAAAGGTG	
rβ-actin	Sense	GTCCACCCGCGAGTACAAC	80
	Antisense	TATCGTCATCCATGGCGAACTGG	
rIL-1	Sense	CCTCGTCCTAAGTCACTCGC	102
	Antisense	GGCTGGTTCCACTAGGCTTT	
rIL-6	Sense	CACTTCACAAGTCGGAGGCT	114
	Antisense	TCTGACAGTGCATCATCGCT	
rα-SMA	Sense	AAGCCCAGCCAGTCGCTGTCA	115
	Antisense	GAAGCCGGCCTTACAGAGCCC	
rTIMP1	Sense	CTTGGTTCCCTGGCGTACTC	150
	Antisense	ACCTGATCCGTCCACAAACAG	
rColl-1	Sense	TGATGGGATTCCCTGGACCT	172
	Antisense	GGGCCTTGTTCACCTCTCTC	
rTNF	Sense	CATCCGTTCTCTACCCAGCC	146
	Antisense	AATTCTGAGCCCGGAGTTGG	
rHGF	Sense	AAAGGGACGGTATCCATCACT	96
	Antisense	GCGATAGCTCGAAGGCAAAAAG	
hBCL6B	Sense	GCTTTGTACAGGTGGCACATC	120
	Antisense	GAACGTGGCTCTTGAGGGTC	
hα-SMA	Sense	TTCATCGGGATGGAGTCTGCTGG	141
	Antisense	TCGGTCGGCAATGCCAGGGT	
hβ-actin	Sense	CTTAGTTGCGTTACACCCTTTC	151
	Antisense	CACCTTCACCGTTCCAGTTT	
hHGF	Sense	GCTATCGGGGTAAAGACCTACA	99
	Antisense	CGTAGCGTACCTCTGGATTGC	
